# In Vitro versus Cryo-Induced Capacitation of Bovine Spermatozoa, Part 2: Changes in the Expression Patterns of Selected Transmembrane Channels and Protein Kinase A

**DOI:** 10.3390/ijms232314646

**Published:** 2022-11-24

**Authors:** Filip Benko, Veronika Fialková, Jana Žiarovská, Michal Ďuračka, Norbert Lukáč, Eva Tvrdá

**Affiliations:** 1 Institute of Applied Biology, Faculty of Biotechnology and Food Sciences, Slovak University of Agriculture in Nitra, Tr. A. Hlinku 2, 949 76 Nitra, Slovakia; 2 AgroBioTech Research Centre, Slovak University of Agriculture in Nitra, Tr. A. Hlinku 2, 949 76 Nitra, Slovakia; 3 Institute of Plant and Environmental Sciences, Faculty of Agrobiology and Food Resources, Slovak University of Agriculture in Nitra, Tr. A. Hlinku 2, 949 76 Nitra, Slovakia; 4 Institute of Biotechnology, Faculty of Biotechnology and Food Sciences, Slovak University of Agriculture in Nitra, Tr. A. Hlinku 2, 949 76 Nitra, Slovakia

**Keywords:** capacitation, cryopreservation, spermatozoa, bull, CatSper, Na^+^/HCO_3_^−^ co-transporter, protein kinase A

## Abstract

Since the molecular similarities and differences among physiological capacitation and cryocapacitation have not been studied in detail, this study was designed to assess the gene and protein expression levels of the Cation channel of sperm (CatSper) 1 and 2, sodium bicarbonate (Na^+^/HCO_3_^−^) cotransporter (NBC) and protein kinase A (PKA) in un-capacitated (control), in vitro capacitated (CAP) and cryopreserved (CRYO) bovine spermatozoa. All samples were subjected to motility evaluation using the computer assisted sperm analysis and chlortetracycline (CTC) assay for the assessment of the capacitation patterns. Furthermore, quantitative reverse transcription PCR (qRT-PCR) and Western blots were used to monitor the expression patterns of the selected capacitation markers. The results showed a significant reduction in the gene and protein expression levels of CatSper1 and 2 in the CRYO group when compared to the CAP group (*p* < 0.0001). In the case of NBC, the results were not significantly different or were inconclusive. While a non-significant down-regulation of PKA was found in the CRYO group, a significant reduction in the expression of the PKA protein was found in frozen-thawed spermatozoa in comparison to the CAP group (*p* < 0.05). In conclusion, we may hypothesize that while in vitro capacitated and cryopreserved spermatozoa exhibit CTC-patterns consistent with capacitation events, the molecular machinery underlying CTC-positivity may be different.

## 1. Introduction

Cryopreservation of ruminant spermatozoa is a powerful tool of the modern livestock industry and cattle production. Despite all advantages, sperm cells are often exposed to stress conditions such as cold shock, formation of ice crystals and osmotic or oxidative pressure. All these stressors may come along with a negative impact on the integrity of the sperm plasmatic membrane. Accumulation of cellular injuries during and after cryopreservation may affect normal cell physiology, leading to a poor fertility potential and a reduced quality of frozen-thawed spermatozoa used for artificial insemination [1,2,3]. A major problem associated with the loss of membrane integrity lies in the reorganization and destabilization of the membrane lipid architecture, including the loss of cholesterol and protein denaturation. Such affected cells are more vulnerable to premature capacitation, spontaneous acrosome reaction and irreversible membrane collapse [4,5].

Capacitation-like changes or cryocapacitation may be described as an early relocation of phospholipids and cholesterol leaching from the plasma membrane, as well as premature consumption of energy generated by the mitochondrial respiratory chain directly after the thawing procedure, which may lead into the loss of sperm movement [6,7,8].

Ion channels are an integral part of the sperm plasma membrane that can be classified into voltage-gated channels such as sodium, potassium and calcium channels, or channels gated by second messengers. These channels are responsible for the regulation of membrane potential, osmotic balance, intracellular pH, and ion concentration [9]. Several critical events occurring in the sperm cell, including hyperactivation and capacitation, depend on calcium (Ca^2+^) ion channels. Different types of voltage-gated Ca^2+^ ion channels are localized in the testis; however, the primary channels present in spermatozoa are the cation channels of sperm (CatSper) [10]. 

In general, the CatSper channel complex is composed of four α sub-units (CatSper 1-4) and six auxiliary sub-units—CatSper β (beta), γ (gamma), δ (delta), ζ (zeta), ε (epsilon) and EFCAB9 (EF-hand calcium-binding domain-containing protein 9)—located in the principal piece of the sperm flagellum in the form of quadrilateral longitudinal nanodomains, which play a pivotal role in the sperm motility. The CatSper complex is responsible for the induction of membrane depolarization on the acrosome and the subsequent influx of Ca^2+^ necessary for flagellar movement, leading to hyperactivated motility [11,12,13,14]. 

During capacitation, members of the sodium bicarbonate cotransporter (NBC) family, which consist of at least four isoforms, are responsible for the transport of Na^+^ (sodium) and HCO_3_^−^ (bicarbonate), cytoplasmatic alkalization, membrane hyperpolarization, regulation of pH and initial anion entrance. Moreover, NBC cotransporters are necessary for the activation of the cAMP/PKA pathway (cyclic adenosine 3′,5′ monophosphate/ protein kinase A) by the uptake of HCO_3_^−^ and subsequent stimulation of sAC (soluble adenylyl cyclase) for the synthesis of cAMP, which will then activate PKA [15,16]. 

The activation of protein kinase A (PKA) leads into actin polymerization through the stimulation of PI3K (phosphatidylinositol 3-kinase). PKA is a tetrameric enzyme containing two R (regulatory) and two C (catalytic) sub-units belonging to the group of S/T (serin/threonine) kinases localized in the acrosomal cap and sperm tail. The enzymatic activity of S/T kinases depends on the concentration of cAMP, which acts as a second messenger and indirectly regulates protein tyrosine phosphorylation. These kinases participate in various sperm functions, such as regulation of ion channels, capacitation, acrosome reaction and flagellar movement [17,18]. 

As mentioned earlier, ion channels, cotransporters and enzymes play a decisive role in the process of mammalian sperm capacitation. In our previous study, we observed that cryopreservation can induce capacitation-like changes in bovine spermatozoa [19]. Cryogenic temperature accompanied with cryocapacitation significantly decreased the quality of post-thawed bovine spermatozoa at the level of their functional activity, structural integrity and oxidative profile. However, the impact of cryopreservation and possible damage associated with cryo-induced capacitation on the level of gene expression and protein profile have still not yet been elucidated enough. 

Hence, the purpose of our study was to investigate the differences in the gene expression of selected genes (*CatSper1*, *CatSper2*, *NBC*, *PKA*), which are directly involved in capacitation events, as well as to assess any possible changes in their protein levels in in vitro capacitated and cryopreserved bovine spermatozoa. 

## 2. Results

### 2.1. Sperm Motility 

**Figure 1:**
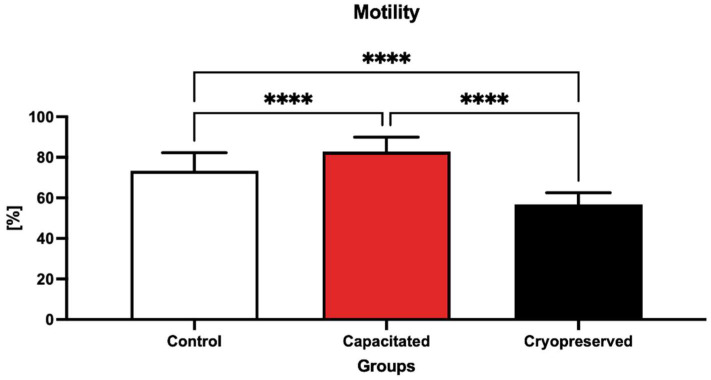
Proportion of motile spermatozoa (%) in the control, in vitro capacitated and cryopreserved group. Each bar represents the motility of bovine spermatozoa in each group (±SD), and the results were obtained by comparing the groups with each other. **** *p* < 0.0001.

The data shown in Figure 1 depict the percentage (%, MOT) of motile spermatozoa in the pre-established groups. The motility significantly decreased (*p* < 0.0001) in the cryopreserved group (56.76 ± 5.77%) when compared to the capacitated group (82.79 ± 7.15%) as well as the control group (73.36 ± 8.94%). Differences in sperm motility were also observed in the capacitated group, where the proportion of motile spermatozoa was statistically increased (*p* < 0.0001) against the cryopreserved and control groups. 

### 2.2. Capacitation Status

**Figure 2:**
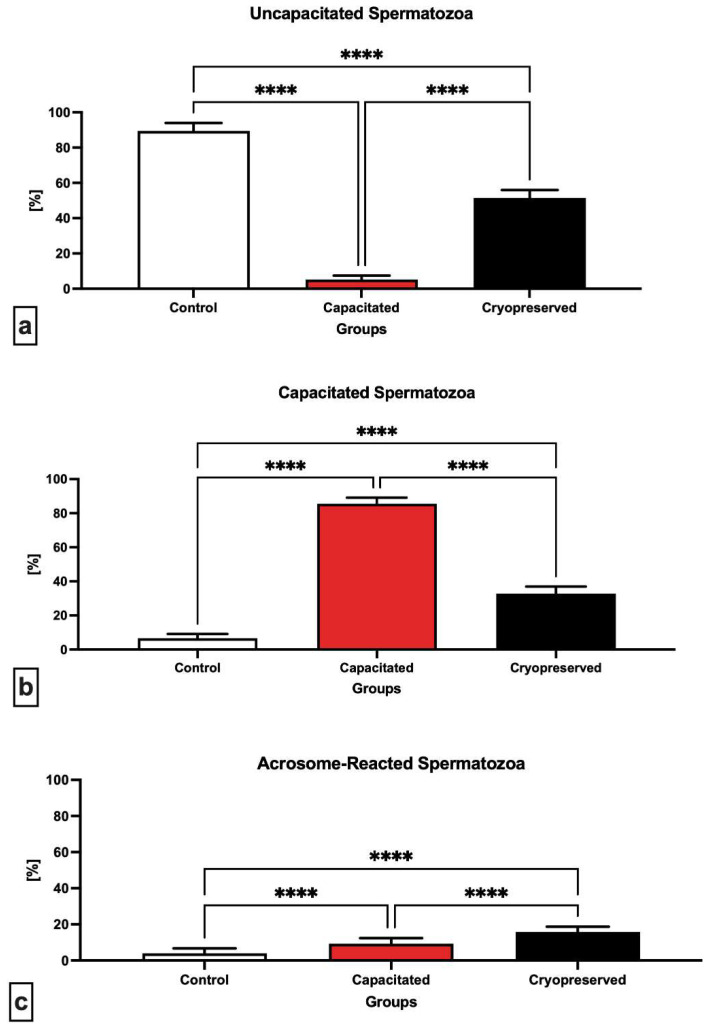
Proportion of un-capacitated (**a**), capacitated (**b**) and acrosome-reacted (**c**) spermatozoa (%) in the control, in vitro capacitated and cryopreserved groups. Each bar represents a CTC staining pattern of bovine spermatozoa in each group (±SD) and the results were obtained by comparing the groups with each other. **** *p* < 0.0001.

Microscopic evaluation of chlorotetracycline (CTC) patterns (Figure 2) indicates the progress of sperm capacitation in the pre-established groups. When compared to the capacitated (5.11 ± 0.93%) and cryopreserved (51.43 ± 4.44%) groups, a significantly higher (*p* < 0.0001) percentage of un-capacitated spermatozoa (“F”-pattern) was found in the control group (89.43 ± 4.51%). Significant differences (*p* < 0.0001) were also observed between the capacitated and cryopreserved group, with an increase in un-capacitated spermatozoa in the cryopreserved group against the capacitated one (Figure 2a).

In the case of cells which underwent capacitation (“B”-pattern), the capacitated group showed a significantly higher (*p* < 0.0001) percentage of capacitated cells (85.57 ± 3.54%) against both residual groups (Figure 2b). However, a significant increase (*p* < 0.0001) in the presence of capacitated cells was also observed in the cryopreserved group (32.81 ± 4.13%) when compared to the control (6.59 ± 0.43%). 

A significant increase (*p* < 0.0001) in acrosome-reacted spermatozoa (“AR”-pattern) was visible in the cryopreserved experimental group (15.76 ± 2.96%) in comparison to the capacitated group (9.31 ± 0.62%) as well as the control group (3.97 ± 0.47%), (Figure 2c). 

### 2.3. Gene Expression Patterns

**Figure 3:**
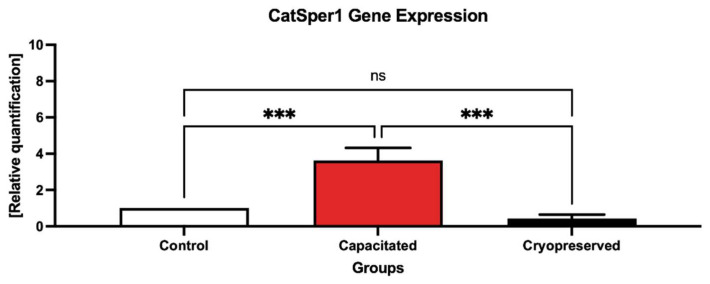
Expression of the *CatSper1* gene in the control, in vitro capacitated and cryopreserved bovine spermatozoa. The results are represented through relative data quantification (±SD) against the control. *** *p* < 0.001. ns—non-significant.

**Figure 4:**
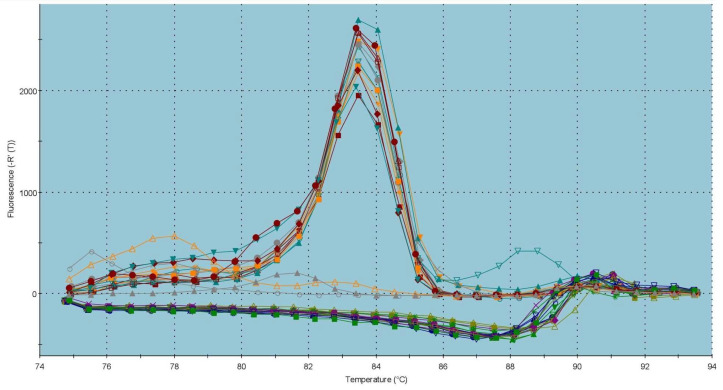
Graphical representation of melting temperatures of amplicons for the *CatSper1* gene. Each colored line with a peak represents one sample analyzed by quantitative reverse transcription PCR (qRT-PCR). Flat lines represent ROX (carboxy-X-rhodamine; ELIZABETH PHARMACON, spol. s r.o., Czech Republic) used as a passive reference dye in ROX-dependent PCR systems to normalize fluorescence intensity of reporter dye in quantitative PCR (qPCR).

The graph on the *CatSper1* gene expression (Figure 3) reveals a significant overexpression of *CatSper1* (*p* < 0.001) in the capacitated group in comparison with the control, which was accompanied by a statistical decrease (*p* < 0.001) in the *CatSper1* gene expression in the cryopreserved group when compared to the control group. The dissociation curve (Figure 4) of amplicon detected a melting peak with a melting temperature (Tm) of 83 °C for *CatSper1*, indicating a specific and unique product.

**Figure 5:**
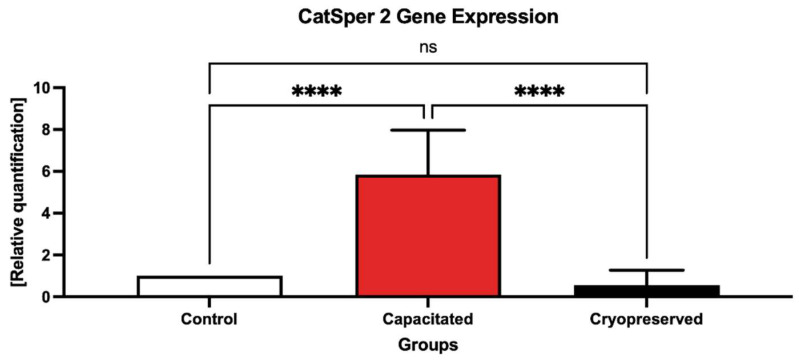
Expression of the *CatSper2* gene in the control, in vitro capacitated and cryopreserved bovine spermatozoa. The results are represented through relative data quantification (±SD) against the control. **** *p* < 0.0001. ns—non-significant.

**Figure 6:**
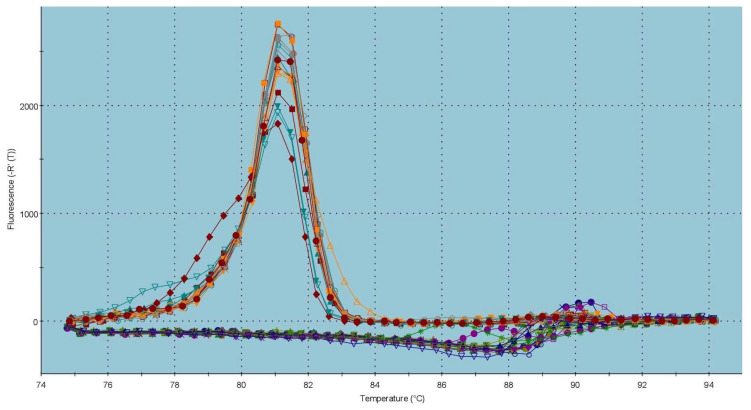
Graphical representation of melting temperatures of amplicons for the *CatSper2* gene. Each colored line with a peak represents one sample analyzed by qRT-PCR. Flat lines represent ROX used as a passive reference dye in ROX-dependent PCR systems to normalize fluorescence intensity of reporter dye in qPCR.

According to the data on the *CatSper2* gene expression, which are shown in Figure 5, the same phenomenon was observed as in the case of CatSper1. A significant overexpression (*p* < 0.001) of *CatSper2* was recorded in the capacitated group against the control. In the meantime, the gene expression of *CatSper2* was statistically decreased (*p* < 0.001) in the cryopreserved group in comparison with the control group. The melting peak of the dissociation curve (Figure 6) indicates a Tm of 81 °C for *CatSper2*. 

**Figure 7:**
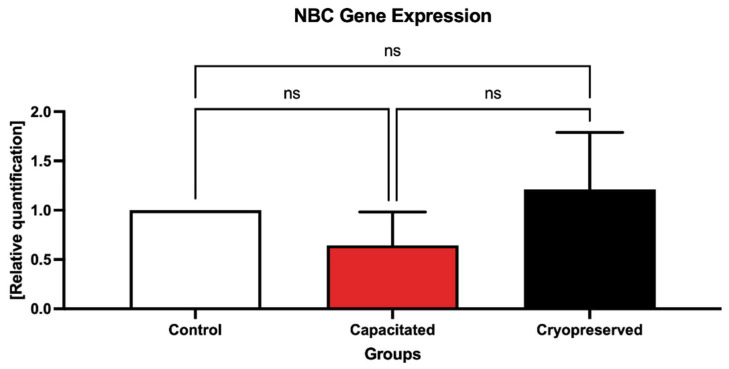
Expression of the *NBC* gene in the control, in vitro capacitated and cryopreserved bovine spermatozoa. The results are represented through relative data quantification (±SD) against the control. ns—non-significant.

**Figure 8:**
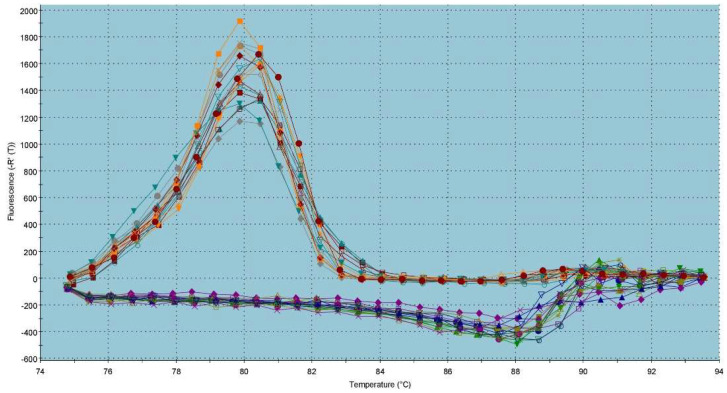
Graphical representation of melting temperatures of amplicons for the *NBC* gene. Each colored line with a peak represents one sample analyzed by qRT-PCR. Flat lines represent ROX used as a passive reference dye in ROX-dependent PCR systems to normalize fluorescence intensity of reporter dye in qPCR.

In the case of *NBC*, a slightly higher gene expression was detected in the cryopreserved group (Figure 7). However, no statistical changes were detected between the pre-established groups. The melting peak for *NBC* within the dissociation curve was visible at a Tm of 80 °C (Figure 8).

**Figure 9:**
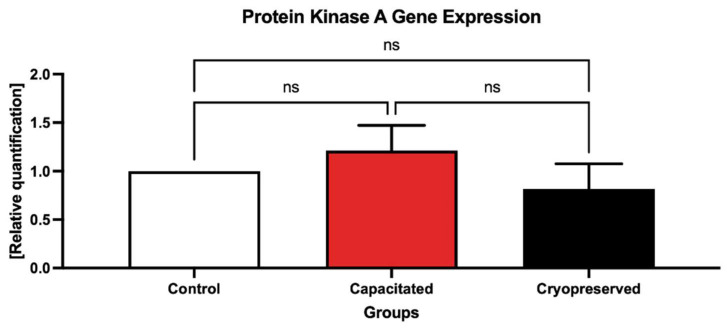
Expression of the *PKA* gene in the control, in vitro capacitated and cryopreserved bovine spermatozoa. The results are represented through relative data quantification (±SD) against the control. ns—non-significant.

**Figure 10:**
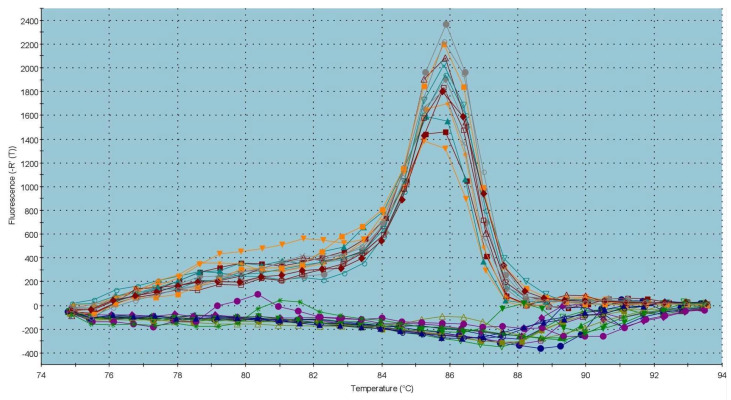
Graphical representation of melting temperatures of amplicons for the *PKA* gene. Each colored line with a peak represents one sample analyzed by qRT-PCR. Flat lines represent ROX used as a passive reference dye in ROX-dependent PCR systems to normalize fluorescence intensity of reporter dye in qPCR.

Evaluation of the *PKA* gene expression (Figure 9) indicated a slight upregulation of the gene in the capacitated group; however, similarly to *NBC*, there was no significant difference between the control and experimental groups. The dissociation curve illustrates a melting peak for *PKA* at a Tm of 86 °C (Figure 10). 

### 2.4. Protein Expression Patterns

**Figure 11:**
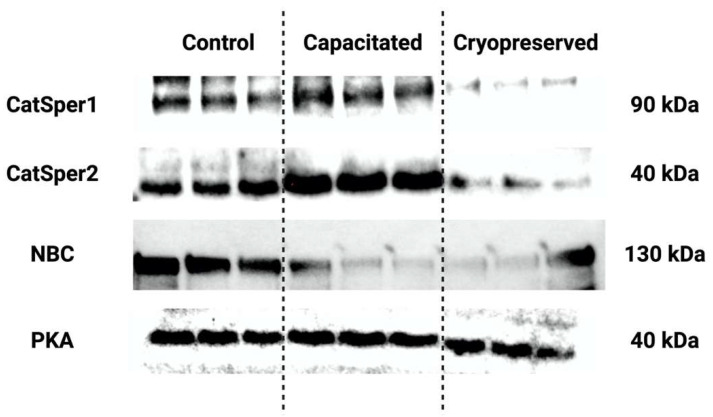
Protein levels of CatSper1, CatSper2, NBC and PKA in un-capacitated control, in vitro capacitated and cryopreserved bovine spermatozoa, as determined by Western blotting. The proteins were separated on 4–15% Mini-PROTEAN TGX Stain-Free Protein Gels (BioRad, Hercules, CA, USA). The loading uniformity was confirmed prior to the blotting procedure using the ChemiDoc Imaging System (BioRad, BioRad, Hercules, CA, USA). Respective bands were visualized using appropriate antibodies and ECL-based chemiluminescence. Precision Plus Protein marker (BioRad, Hercules, CA, USA) was used on each gel to indicate the molecular weight of the separated proteins. Original photos of the gels, membranes and blots are available as Supplementary Materials. Created with (Supplementary: Confirmation of Publication and Licensing Rights) BioRender.com (accessed on 28 September 2022).

Protein levels of CatSper1, CatSper2, NBC and PKA in the control, in vitro capacitated and cryopreserved spermatozoa, as determined by Western blotting, are depicted in Figure 11.

**Figure 12:**
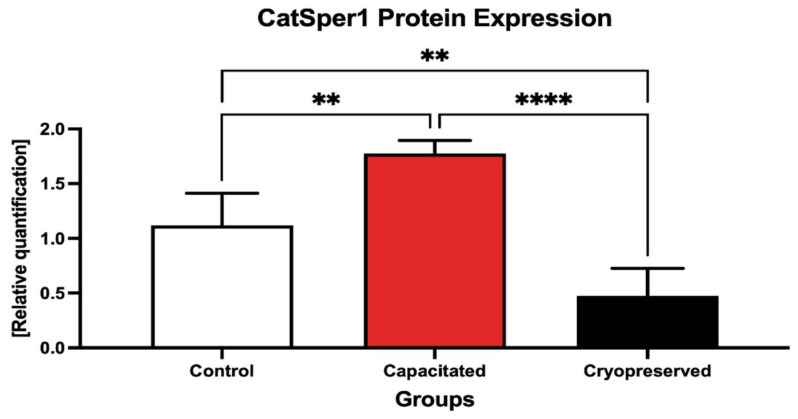
Expression of the CatSper1 protein in the control, in vitro capacitated and cryopreserved bovine spermatozoa. The results are represented through relative data quantification (±SD) against the control. ** *p* < 0.01; **** *p* < 0.0001.

The Western blot analysis revealed that the CatSper 1 protein was significantly up-regulated in in vitro capacitated spermatozoa in comparison to the control (*p* < 0.01, Figure 12, Figures S3 and S4). Inversely, a significant down-regulation of the protein was observed in the cryopreserved groups when compared to both the control (*p* < 0.01) and the capacitated group (*p* < 0.0001).

**Figure 13:**
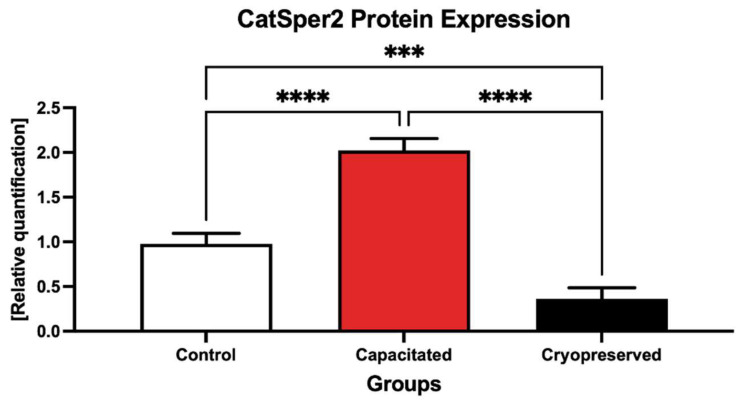
Expression of the CatSper2 protein in the control, in vitro capacitated and cryopreserved bovine spermatozoa. The results are represented through relative data quantification (±SD) against the control. *** *p* < 0.001; **** *p* < 0.0001.

CatSper2 seemed to respond in an even more pronounced manner to changes induced in the experimental groups (Figure 13, Figures S5 and S6). A significantly higher expression of the protein was recorded in in vitro capacitated spermatozoa when compared to the control (*p* < 0.001). On the contrary, a significant decline in the protein was noted in the cryopreserved group in comparison with the control (*p* < 0.0001), as well as the in vitro capacitated group (*p* < 0.0001). 

**Figure 14:**
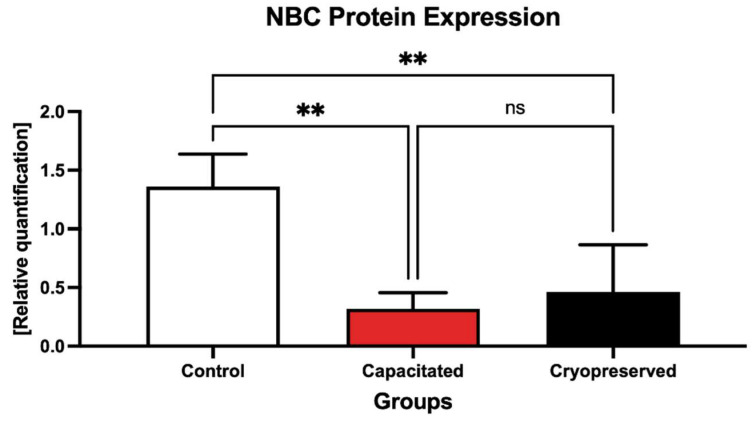
Expression of the NBC protein in the control, in vitro capacitated and cryopreserved bovine spermatozoa. The results are represented through relative data quantification (±SD) against the control. ** *p* < 0.01. ns—non-significant.

NBC protein expression was found to be affected by the process of both in vitro capacitation cryopreservation (Figure 14, Figures S7 and S8). A significantly decreased level of NBC was found in both the capacitated and the cryopreserved groups when compared to the control (*p* < 0.01).

**Figure 15:**
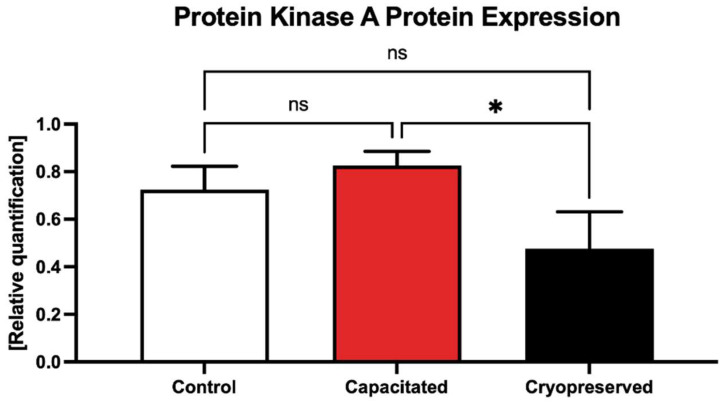
Expression of the PKA protein in the control, in vitro capacitated and cryopreserved bovine spermatozoa. The results are represented through relative data quantification (±SD) against the control. * *p* < 0.05. ns—non-significant.

When comparing the protein levels of PKA, no significant changes were detected when comparing the control with in vitro capacitated spermatozoa, although PKA expression levels were higher in the capacitated group (Figure 15, Figures S9 and S10). A significant under-expression of PKA was detected in the cryopreserved group in comparison with the un-capacitated control (*p* < 0.05).

## 3. Discussion

Sperm capacitation encompasses a series of complex biochemical and morphological processes that take place in the female reproductive tract, and which are crucial for the sperm cell to successfully reach the oocyte, bind to the zona pellucida, and fuse with the oolemma. Biochemical changes in the plasma membrane and other subcellular compartments have been linked to sperm capacitation [20]. Major molecular events associated with the capacitation process involve, among others, the activation of calcium channels with a subsequent alkalinization of the sperm cell, reactive oxygen species (ROS) generation (particularly superoxide and hydrogen peroxide) [8,21], cAMP synthesis and the activation of protein kinases, accompanied by cholesterol efflux from the sperm plasma membrane [22]. 

A major consequence of the events associated with the capacitation process is sperm hyperactivation, which may be observed as a significant increase in the sperm motility, as observed in our study. This is in accordance with earlier reports [23,24,25,26]. It has been previously noted that hyperactivated spermatozoa swim two to three times faster than their uncapacitated counterparts, since the tail beat switches from a sinusoidal pattern to a wide tail bending and a major head oscillation [23]. This behavior is necessary for the sperm to detach itself from the fallopian villi when ovulation occurs, as well as to enable the sperm cell to meet the oocyte and to enable the acrosome reaction [20,26,27]. Inversely, studies on diverse farm animals have repeatedly observed a significantly compromised motion behavior of frozen-thawed spermatozoa (reviewed by [1,7]), which has been recorded in our experiments as well. The causes for the motility loss of male gametes following cryopreservation are diverse. The primary reason may lie in compromised mitochondrial function [27,28] or greater-than-optimal ROS concentrations (in particular, superoxide and peroxides) with subsequent oxidative damage to molecules vital for the sperm activity [29]. Furthermore, damage to the plasma membrane as a result of ice crystals and lipid peroxidation can affect motility patterns, causing sperm to have different patterns of movement, which may affect the resulting fertilization ability of spermatozoa [30].

Despite changes observed in the motility behavior of spermatozoa in the pre-established groups, it has been previously postulated that cryopreserved sperm appear to undergo changes consistent with premature capacitation. In this sense, it may be stated that although freeze-thawed spermatozoa are able to accomplish fertilization, their life span following artificial insemination (AI) is notably shorter in comparison to freshly ejaculated spermatozoa that have not yet undergone capacitation [31,32]. As indicated by De Leeuw et al. [33], as well as by Holt and North [34], temperature oscillations may induce a lipid phase change from a liquid-crystalline to gel phase, leading to an increased membrane fluidity. This process may be accompanied by phosphatidylserine translocation and expulsion or relocation of membrane proteins and phospholipids. This may resemble the events which occur during a physiological capacitation [35]. This series of changes may be responsible for the membrane of cryopreserved spermatozoa to respond to the chlortetracycline (CTC) stain, which has been observed in our experiments. Similar CTC staining patterns were observed in both capacitated and cryopreserved sperm in bulls [19,36], boars [37,38], stallions [39] and rams [40].

While some common aspects of capacitation and cryocapacitation have previously been indicated, the molecular mechanisms linking or distinguishing both events are currently not well-understood. As such, in this study, we compared genetic and protein expression patterns of two major transmembrane channels and one enzyme known to play a vital role in the process of capacitation in in vitro capacitated and cryopreserved bovine spermatozoa.

CatSper channels are highly evolutionary conserved calcium ion channels, present exclusively in the male gamete, which regulate the transport of Ca^2+^ into the intracellular environment and contribute to the acquisition of hyperactivated sperm motility and fertilization ability [41]. Up-regulation of the *CatSper1* and *2* subunits on a gene, as well as the protein levels observed in the in vitro capacitated group, which were pre-established in our experiments, support this theory and complement the increase in sperm motility detected by the CASA analysis. These results may agree with earlier studies, in which CatSper channels were activated by progesterone, zona pellucida glycoproteins, cyclic nucleotides or bovine serum albumin (BSA) in human [42,43], mouse [44,45] and Rhesus monkey sperm [46], leading to a hyperactivated motion behavior and a successful acrosome reaction and/or fertilization. Overexpression of both genes and proteins during capacitation further confirms their undeniable role in achieving the fertilization ability of male gametes, since men presenting with *CatSper1* or *CatSper2* mutations are infertile [47,48,49] and null mice deprived of any of CatSper isoforms are unable to reproduce [50,51]. This is because CatSper-deficient sperm cells do not hyperactivate, and, thus, fail to penetrate the *cumulus oophorus* of the oocyte [11]. 

On the other hand, an increase in the production of *CatSper1* messengers has been previously observed in mice, suggesting that the mRNA (messenger RNA) is located in the middle piece and the sperm head, so the translation seems to be carried out by the mitochondrial translational machinery [52]. Since an increased CatSper protein expression has been recorded in this study, which employs bovine spermatozoa, it seems that there are similar patterns of *CatSper* messengers among mammals. Hence, it would be interesting to study this pathway in other animal models, such as rams, bucks or stallions, to confirm or exclude possible similarities and differences.

Nevertheless, as emphasized by Darszon et al. [53], voltage-gated calcium channels (Cav) also contribute to Ca^2+^ influx into the spermatozoon, which is why their expression patterns could shed more light into their contribution to reach critical Ca^2+^ levels necessary for hyperactivation to occur. Furthermore, measurement of the oscillation in Ca^2+^ concentration prior, during and following capacitation could add more information to the exact dynamics of Ca^2+^ entry to the male gamete during physiological capacitation.

During the freeze and thaw procedures, male gametes are exposed to potentially detrimental physico-chemical phenomena, such as the formation and dissolution of intracellular ice crystals. These may affect the membrane permeability and cause intracellular dehydration or osmotic injury [54]. The results of this study reveal decreased expression of CatSper1 and 2 in the cryopreserved samples, in comparison to both the control and in vitro capacitated spermatozoa. This observation is in agreement with Alshawa et al. [55], who found a significant *CatSper2* mRNA down-regulation in cryopreserved human spermatozoa when compared to fresh semen. As speculated by Flores et al. [56], such a decrease in the gene transcription and translation may be linked to alterations in mRNA–protein interactions and a higher susceptibility of mRNA to degradation due to cryopreservation. A different hypothesis on the loss of membrane proteins during the freeze–thaw process was postulated by Harrison et al. [57], according to whom transmembrane proteins could be leaking to the extracellular surroundings or the semen extender as a response to cold shock. In this sense, it may be feasible to assess any trace of CatSper domains in the cryopreservation medium, although Zhang et al. [58,59] tried to verify this theory in the case of heat shock proteins without any success. Another possibility to explain such a significant down-regulation of CatSper may be connected to the fact that frozen-thawed sperm samples often present with an increased number of already dead cells, which are unable to produce any protein anymore [60]. Finally, a possible cytotoxicity of the cryoprotectant may play a role in any alterations to the sperm plasma membrane and adjacent molecular components. Previous papers have also reported a significant decrease in proteins such as tektin, vimentin, aconitase 2 or enolase 1, all of which are known to be intricately involved in the regulation of sperm motility, membrane and acrosome integrity, Ca^2+^ transport and capacitation events [55,61,62]. As such, we may agree with a growing body of evidence indicating that sperm dysfunction following cryopreservation may be at least partially associated with protein degradation and subsequent loss of function. 

Numerous earlier reports have revealed that capacitation is dependent on HCO_3_^−^, since its transmembrane fluctuations have been associated with increased intracellular pH values and subsequent stimulation of adenylyl cyclase, which is responsible for cAMP synthesis [63,64,65]. Further experiments have unraveled the presence of several transmembrane HCO_3_^−^ channels in rat [66] and mouse sperm [16], out of which NBC seems to be the predominant one in the regulation of sperm plasma membrane hyperpolarization and additional events leading to the capacitation state.

In this sense, an overexpression of the *NBC* gene would have been expected; nevertheless, this was not confirmed by our RT-PCR analysis. In fact, we recorded a moderate under-expression of *NBC*. The reasons behind this observation are subject to speculation, since the mechanisms of *NBC* activity regulation during capacitation are not clear yet. 

According to Visconti [67], sperm capacitation may be characterized by slow and fast events. During the initiation of the fast events, it seems that the primary molecular component to be activated is precisely NBC, which will then stimulate CatSper channels with a subsequent influx of Ca^2+^, cAMP synthesis and PKA activation, culminating in motility activation. During slow events occurring during a prolonged period of sperm exposure to a capacitation-stimulating environment, the sperm cell gains its ability to fertilize, which is accompanied by hyperactivation. These events are defined by a rather steady influx of HCO_3_^−^, necessary for a continuous production of cAMP, stabilization of PKA activity and tyrosine phosphorylation. Since we did observe CatSper overexpression and a hyperactivated motility alongside a CTC positivity, we may assume that we might have collected the cells for RNA and protein harvesting during slow capacitation events, when NBC may not be as active as it is during the very first stages of the capacitation process. This may be in agreement with Gadella and Harrison [35], hypothesizing that cholesterol efflux could be involved in the regulation of NBC; thus, its activity could decrease following membrane reorganization and the loss of cholesterol. Nevertheless, since a steady flow of sodium (Na^+^), and particularly HCO_3_^−^, is necessary to successfully accomplish capacitation, we may speculate that other Na^+^ or HCO_3_^−^ transmembrane exchangers may become involved, including SLC26 transporters responsible for HCO_3_^−^/Cl^−^ regulation or the sperm Na^+^/H^+^ exchanger (sNHE) [67,68]. As such, it would be highly feasible to assess their expression dynamics in un-capacitated as well as capacitated spermatozoa. Furthermore, a parallel measurement of the intra- and extracellular fluctuations of HCO_3_^−^ and Na^+^ could shed more light on the dynamics of the transmembrane channels responsible for the influx of anions during capacitation events.

A different issue lies in the interpretation of the data collected from the Western blot analysis. While a strong chemiluminescent signal was detected in case of the control group involving by and large un-capacitated spermatozoa, a very weak signal was observed in both experimental groups. Hence, we may speculate that the activation of the NBC protein, either by an environment favoring physiological capacitation or by low temperatures, most likely causes changes in the structure or conformation of the channel, which will not be detectable by the antibodies used in our experiments. According to Molina et al. [15], the NBC family consists of at least four isoforms with different stoichiometries. As such, we may assume that stimulation of the NBC channel may cause structural changes, reorganization of its subunits or a possible switch from an electroneutral isoform to an electrogenic one, which may not be detected by the same antibody. Verification of this hypothesis should, nevertheless, be the subject of further studies.

As opposed to oocytes or embryos, male gametes have a very limited capacity to maintain the stability of intracellular pH [69], which is why the seminal plasma in fresh ejaculates acts as an important buffer, keeping the pH of the semen close to neutral values, ranging from 7.2 to 8.2. Yet, during sperm storage, the pH decreases gradually due to accumulation of acidic metabolites. It is generally accepted that acidic environments are toxic to spermatozoa, rendering them immobilized [70,71]. Since HCO_3_^−^ acts as a natural buffering agent of semen, we may hypothesize that NBC overexpression in cryopreserved spermatozoa may only partially be associated with premature capacitation. Since NBC is responsible for an increased influx of HCO_3_^−^, its elevated expression patterns in cryopreserved spermatozoa in comparison to their in vitro capacitated counterparts could indicate its involvement in the sperm defense mechanisms against the loss of motility due to low pH. On the other hand, this abnormal overstimulation became a starting point to a general ionic imbalance within the sperm cell, possibly initiating changes associated with cryocapacitation. Nevertheless, similarly to in vitro capacitated spermatozoa, the protein expression of NBC was very low. This may lead us to speculate that activation of NBC channels, either because of changes associated with premature capacitation or alkalization, leads to the same structural changes in the channel, which are difficult for the antibodies used in this study to detect. Finally, since NBC is a transmembrane channel, its low protein expression may, at least partially, be caused by its loss due to membrane damage, as observed in the case of the CatSper proteins.

Protein kinase A (PKA) is a broad-spectrum tetrameric serine/threonine protein kinase involved in the regulation of several cellular activities [72]. Once activated by cAMP, the catalytic subunits of the enzyme phosphorylate a broad variety of substrates in the Arginine (Arg)-X-X-Serine/Threonine motifs (X represents any amino acid) [73,74]. Within spermatozoa, PKA activity is primarily associated with protein tyrosine phosphorylation, actin polymerization, and the development of hyperactivated motility [75]. This indisputable role of PKA has been previously confirmed by Nolan et al. [76], according to whom mice sperm that lack the PKA catalytic subunit Cα2 are infertile, despite normal mating behavior, and do not accomplish tyrosine phosphorylation despite being exposed to it in a capacitation medium. The involvement of PKA in the capacitation events has been furthermore corroborated by studies, in which treatments with inhibitors of adenylyl cyclase and/or PKA resulted in a reduction in sperm motility initiation [67,77] and prevention of the acrosome reaction [78,79]. 

Consistently with our results, Visconti et al. [80] reported that PKA activity progressively increased in mouse epididymal spermatozoa during capacitation. Furthermore, Lefièvre et al. [18] observed that PKA activity was higher in capacitated as opposed to un-capacitated human spermatozoa throughout 3 h of incubation in fetal cord serum ultrafiltrate. Similarly to our qRT-PCR analysis, Lee-Estevez et al. [81] observed a slight increase in PKA activity in freshly activated compared to non-activated spermatozoa; yet, there was not a statistically significant difference. We must agree with the authors that considering that PKA activation occurs very rapidly after spermatozoa are exposed to a capacitation-promoting environment, further experiments on a wider selection of post-activation time intervals may provide additional insights into its involvement in motility activation mechanisms. 

More importantly, we observed that the PKA activity was notably reduced in the cryopreserved samples, suggesting that the enzyme may be altered by the cryopreservation procedure, with subsequent effects on downstream signaling events, leading to motility activation. This is in agreement with Lee-Estevez et al. [81], who noted a loss of PKA activity in frozen-thawed fish spermatozoa, which was fortified by a significant positive correlation between PKA activity and percentage of motile spermatozoa. Aside from a direct loss of the enzyme by exposure of spermatozoa to low temperatures, other factors may play a role in the decreased activity of PKA in cryopreserved spermatozoa. As revealed by Chen et al. [82], the proteomic profiling of frozen-thawed boar revealed alterations in the protein expression of A-kinase anchoring proteins (AKAPs), which bind PKA to the cytoskeleton or to sub-cellular organelles in close proximity to target proteins [83]. Furthermore, the anchoring of PKA to AKAP is actively regulated during sperm capacitation. Hence, any alterations to the dynamics of AKAPs may have a direct impact on proper PKA anchoring, and, subsequently, the extent by which PKA phosphorylates target proteins in the sperm cell. Finally, as reported by Stival et al. [72], altered PKA anchoring may lead to a disruption in its localization within the sperm structures, leading to premature acrosome exocytosis, and providing a possible explanation for the increased proportion of cells exhibiting positive CTC “AR” patterns in the cryopreserved group.

The results of this article reinforce previous observations regarding the presence of mRNA in mature spermatozoa. However, it is difficult to accomplish active transcription in spermatozoa whose genome is highly packaged. We may exclude de novo synthesis, since condensed mature spermatozoa have a significantly reduced cytoplasm, and lack ribosomes that would support de novo translation. As such, we may speculate that RNA and protein synthesis in mature spermatozoa could be mainly driven by mitochondria from paused transcripts waiting for activation, possibly through capacitation or another metabolic pathway. In addition, the decrease in some transcripts following capacitation may be the result of higher translational activity for more protein synthesis, which introduces the theory that capacitation promotes post-translational modifications. 

**Figure 16:**
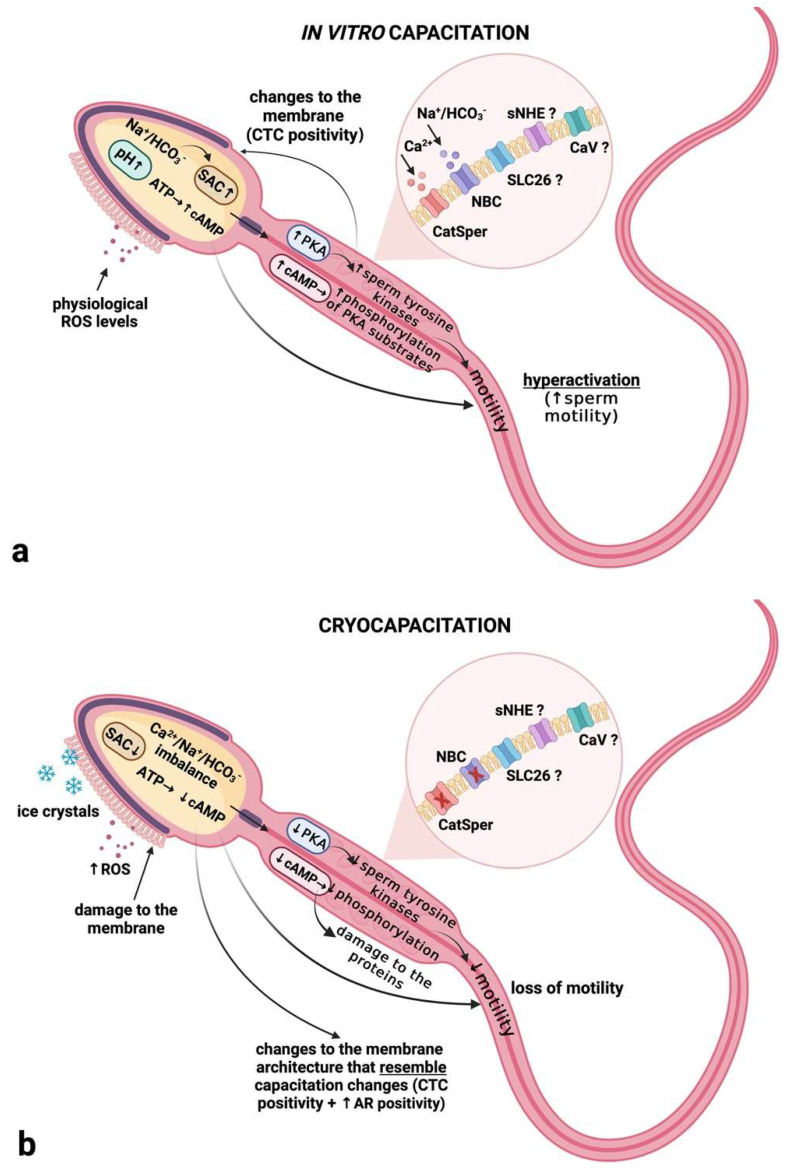
Suggested sequence of events occurring during in vitro capacitation (**a**) and cryocapacitation (**b**). (**a**) Exposure of bovine spermatozoa to the capacitation medium activates the transmembrane channels, leading to an influx of HCO_3_^−^ and Ca^2+^ into the intracellular compartments of the male gamete, as well as an increased pH and activation of soluble adenylyl cyclase (sAC), which will drive the synthesis of cyclic adenosine monophosphate (cAMP) in the presence of physiological levels of reactive oxygen species (ROS). Subsequently, increased cAMP levels will activate protein kinase A (PKA), resulting in increased stimulation of tyrosine kinases and phosphorylation of PKA substrates. This chain of events will then favor hyperactivated sperm motility and changes in the properties of the sperm plasma membrane, visible in the chlortetracycline (CTC) assay as an increase in spermatozoa exhibiting a “B” pattern. The involvement of other transmembrane channels in the changes to the intracellular ionic milieu remains unclear. (**b**) Temperature oscillation and the formation of ice crystals and ROS overproduction, as accompanying side effects of cryopreservation, lead to alterations in the function or to a direct loss of the CatSper and NBC transmembrane channels, as well as to a subsequent imbalance in the intracellular ionic milieu. This phenomenon may lead to a decreased activity level of sAC, followed by a reduced level of cAMP and a lower activity level of PKA, manifesting itself as a decline in post-thaw sperm motility. At the same time, temperature changes may promote the liquid-crystalline phase of the sperm membrane to transit into a gel phase, leading to increased membrane fluidity, accompanied by the loss of membrane proteins and phospholipids, as well as an increased response of the cells to the CTC stain. Furthermore, a possible PKA dislocation may contribute to a premature acrosome reaction and a subsequent increase in the CTC “AR” patterns of cryopreserved spermatozoa. The effect of cryopreservation on other transmembrane channels in the changes to the intracellular ionic milieu remains unclear. Created with (Supplementary: Confirmation of Publication and Licensing Rights) BioRender.com (accessed on 29 September 2022).

To summarize our results, we may assume that cryopreservation induces a cascade of molecular events in spermatozoa, which reveal themselves as structural and functional changes different from those characteristics of un-capacitated spermatozoa or those of capacitated but acrosome-intact cells. Whilst the changes in the CTC staining patterns of frozen-thawed spermatozoa resemble those occurring during a physiological capacitation driven by the influx of HCO_3_^−^, Ca^2+^, cAMP synthesis, PKA activation and protein phosphorylation (Figure 16a), the key channels and enzymes of capacitation are significantly altered by the cryopreservation process (Figure 16b). We may consider our data as preliminary, since further studies on the dynamics of other molecules playing critical roles in the capacitation events are crucial for a better understanding of whether the changes observed after cryopreservation are the same as those seen during in vivo or in vitro capacitation.

## 4. Materials and Methods

### 4.1. Biological Material and Culture 

Semen samples were obtained from 30 healthy adult Holstein Friesian bulls at a local breeding facility (Slovenské biologické služby, a.s., Nitra, Slovakia) with the help of an artificial vagina. All samples (1 sample per bull) were distributed into three fractions. The first and second ones were immediately transported into the laboratory and incubated for 30 min at 39 °C and 5% concentration of CO_2_, either with physiological saline solution (IMUNA PHARM, A. S., Šarišské Michaľany, Slovakia) as a control or with a capacitation medium consisting of 100 mM sodium chloride (Sigma-Aldrich, St. Louis, MO, USA), 3 mM potassium chloride (Sigma-Aldrich, St. Louis, MO, USA), 25 mM sodium bicarbonate (Sigma-Aldrich, St. Louis, MO, USA), 283 µM sodium phosphate (Sigma-Aldrich, St. Louis, MO, USA), 10 mM HEPES (Sigma-Aldrich, St. Louis, MO, USA), 1.5 mM magnesium chloride (Sigma-Aldrich, St. Louis, MO, USA), 2.5 mM calcium chloride (Sigma-Aldrich, St. Louis, MO, USA), 0.37% sodium DL-lactate solution (60%; Sigma-Aldrich, St. Louis, MO, USA) and 0.2% phenol red solution (0.5%; Sigma-Aldrich, St. Louis, MO, USA) [84] diluted in a ratio of 1:40. The third part was cryopreserved for further investigation.

### 4.2. Cryopreservation Procedure

Fractions of the semen samples selected for cryopreservation, with a concentration of 44 × 10^6^ spermatozoa/mL, were prepared and diluted in 20% egg yolk extender. They were then filled into 0.25 mL French straws by using automatic straw filler (Minitüb GmbH, Tiefenbach, Germany), cooled at 4 °C/2 h and frozen with a digital freezer (Digitcool Model 5300 ZB 250; IMV, L’Aigle, France) as follows: −3 °C/min. from +4 °C to −10 °C; −40 °C/min. from −10 °C to −100 °C; −20 °C/min. from −100 °C to −140 °C. Afterward, all straws were transferred into a container filled with liquid nitrogen and stored at −196 °C for at least one month (Slovenské biologické služby, a.s., Nitra, Slovakia). 

### 4.3. Thawing and Washing Procedure 

Cryopreserved straws were thawed using a heating pad at 37 °C for 30 s. Then, the whole volume of each straw was transferred into labeled 0.5 mL tubes, and the proportion of motile spermatozoa was evaluated with the computer-assisted sperm analysis (CASA) system. After that, the samples were washed three times by adding 500 µL of PBS (phosphate saline solution; Sigma-Aldrich, St. Louis, MO, USA) and centrifuged for 10 min/5000 RPM for the disposal of egg yolk extender residues. 

For the extraction of RNA and proteins, the cell suspensions were subjected to a single-layer Percoll^®^ Plus (Sigma-Aldrich, St. Louis, MO, USA) density gradient separation, according to Ďuračka et al. [85].

### 4.4. Computer-Assisted Sperm Analysis

The proportion of motile bovine spermatozoa (%; MOT) was observed with CASA (version 14.0 TOX IVOS II; Hamilton-Thorne Biosciences, Beverly, MA, USA). Ten µL of sample were dropped into the Makler’s counting chamber (depth 10 µm, 37 °C; Sefi Medical Instruments, Haifa, Israel), and the final data were assessed using the Animal Motility program (Hamilton-Thorne, Biosciences, Beverly, MA, USA).

### 4.5. Capacitation Patterns 

**Figure 17:**
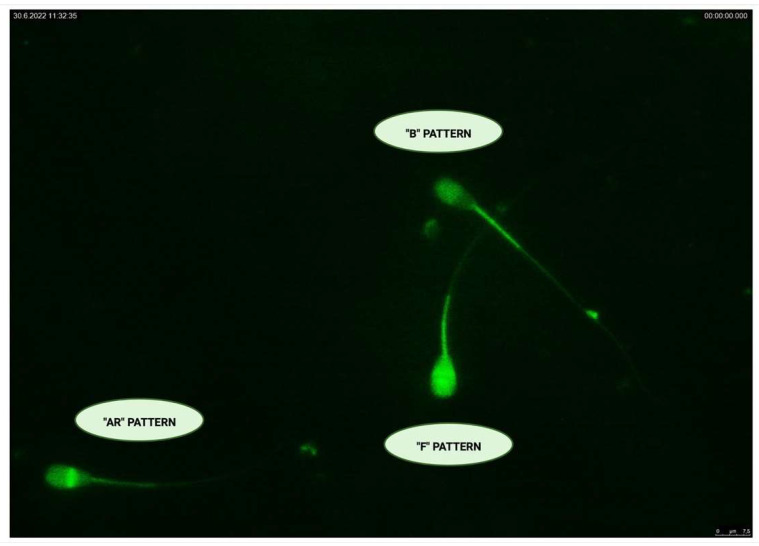
CTC fluorescence patterns in different stages of the capacitation events. Original photograph is available as Supplementary Material. Created with (supplementary: Confirmation of Publication and Licensing Rights) BioRender.com (accessed on 28 September 2022).

The evaluation of capacitation status was performed with chlortetracycline (CTC; Sigma-Aldrich, St. Louis, MO, USA) assay, and identification of specific CTC patterns after absorption of dye by spermatozoa was observed under the Leica DMI6000 B (Leica Camera, Wetzlar, Germany) fluorescent microscope (Figure 17 and Figure S11). According to cell coloration, three basic patterns were identified: fluorescence of the whole sperm head for un-capacitated spermatozoa (“F” pattern), fluorescence of the sperm head except for post-acrosomal region, which is characteristic for capacitated spermatozoa (“B” pattern) and only a bright fluorescent band in the equatorial segment stood for acrosome-reacted spermatozoa (“AR” pattern) [19,86]. Thirty samples from each group were assessed, and at least 300 cells were counted in each sample. 

### 4.6. RNA Isolation and cDNA Synthesis 

The isolation of total RNA was performed using NucleoZOL reagent (MACHEREY-NAGEL GmbH & Co. KG, Düren, Germany) with a slight modification of the manufacture’s protocol for the extraction procedure from bovine spermatozoa. Briefly, a total of 700 mg/µL of frozen cell suspension was homogenized in 1 mL of NucleoZOL for 1 min in order to ensure a complete lysis of spermatozoa. Then, all samples were centrifuged for 15 min/12,000 RPM at 4 °C, and the supernatant was washed with 700 µL chloroform to achieve phase separation. After that, isolation was completed according to the manufacturer’s recommendations.

The quantity and purity of extracted RNA and A260/A280 nm ratios were assessed by Implen NanoPhotometer (München, Germany). Only high-purity RNA (A260/A280 ≥ 2) was used for cDNA synthesis. For the reverse transcription and cDNA synthesis, we used 200 ng of total extracted RNA and the commercial Maxima First Strand cDNA Synthesis Kit for RT-qPCR with dsDNase (ThermoFisher Scientific, Waltham, MA, USA).

### 4.7. Real-Time qPCR 

Primers for real-time qPCR were designed based on the mRNA sequences of selected genes (*CatSper1*, *CatSper2*, *NBC*, *PKA*), and the housekeeping gene GAPDH was used as a reference gene. All primer sequences (Table 1) were designed using the Primer-Blast program (Bethesda, MD, USA).

Specific PCR conditions were optimized for each pair of primers, which included annealing temperature and PCR efficiency standard curve analysis. The specificity of PCR amplification was checked through to end-point PCR and melting-curve analysis.

Quantitative PCR (qPCR) was performed with 5× Elizyme^TM^ Green Mix AddROX (ELIZABETH PHARMACON, spol. s r.o., Brno, Czech Republic) according to the manual’s instructions, using the Stratagene Mx3005P thermocycler (Agilent, Santa Clara, CA, USA) under specific cycling conditions: 95 °C/2 min. then 45 cycles of 95 °C/10 s; 58 °C/40 s (*CatSper1*/*2*), 59 °C/40 s (*PKA*), 60 °C/40 s (*GAPDH*/*NBC*); 72 °C/10 s. All qPCR experiments were performed in triplicate. Dissociation curves were generated at the end of every run to ensure product uniformity, and the size and specificity of final PCR products were verified on 2% agarose gel.

### 4.8. Western Blot 

Proteins from washed-out spermatozoa were used for the Western blot analysis. In order to achieve this, the cells were treated with 1 mL RIPA buffer (Sigma-Aldrich, St. Louis, MO, USA) with protease inhibitor (Sigma-Aldrich, St. Louis, MO, USA) to prevent protein degradation. The samples were thoroughly mixed with the lysis buffer, and subsequently left in the refrigerator overnight. The next day, the samples were mixed again and centrifuged at 11,828× *g* for 10 min at 4 °C. The supernatants were collected for the quantification of total proteins [87].

Protein determination was performed using a photometric test, based on the Biuret reaction. In this assay, a reaction occurs between proteins and copper ions, forming complexes in an alkaline solution and resulting in a blue-violet color. We used a commercial total protein kit (DiaSys, Holzheim, Germany), and the assay was performed on the RX Monza instrument (Randox, Crumlin, UK).

Prior to the Western blot assay, all lysates were normalized, i.e., protein concentration was adjusted using ultrapure (UHQ) water to reach a final concentration of 25 μg protein. The samples were treated with 4× Laemli buffer (BioRad, Hercules, CA, USA) and β-mercaptoethanol (Sigma-Aldrich, St. Louis, MO, USA), and then boiled at 95 °C for 10 min. The pre-treated samples were loaded (20 μL) into Mini-PROTEAN TGX stain-free polyacrylamide gels (BioRad, Hercules, CA, USA), along with 7 μL of Precision Plus Protein marker (BioRad, Hercules, CA, USA). Gel electrophoresis was run at 90 V for 2 h, and subsequently, the gels were visualized with the ChemiDoc Imaging System (BioRad, Hercules, CA, USA) to confirm the loading uniformity (Figure S1). For the blotting procedure, the gels were transferred to PVDF membranes (Trans-Blot Turbo Pack; BioRad, Hercules, CA, USA) using the Trans-Blot Turbo Transfer System (BioRad, Hercules, CA, USA), 25 V, 2.5 A, for 7 min (for CatSper1/2 and PKA) or 10 min (for NBC). After completion of the blot, the sandwich was disassembled, and the membranes were incubated for 3 × 10 min in Tris-buffered saline (TBS), composed of Tris base (Sigma-Aldrich, St. Louis, MO, USA), sodium chloride (Centralchem, Bratislava, Slovakia) and UHQ water. This step was followed by membrane staining with Ponceau S solution (Sigma-Aldrich, St. Louis, MO, USA) to visualize the bands on the membranes. Subsequently, the membranes were cut, following the marker, into smaller pieces, where the protein of interest was presumably localized. The membranes were blocked with 5% skim milk (Blotting grade blocker; BioRad, Hercules, CA, USA) in TBS containing 0.1% Tween-20 (Sigma-Aldrich, St. Louis, MO, USA). Membrane blocking was performed on a stirrer at room temperature for 2 h. Finally, the membranes were incubated with one of the following primary antibodies:

- CATSPER1 Polyclonal Antibody (#PA5-75788; Invitrogen, Waltham, MA, USA), source: rabbit, dilution 1:1000 in 5% milk/TBS/0.1% Tween-20;
- CATSPER2 Polyclonal Antibody (#PA5-41072; Invitrogen, Waltham, MA, USA), source: rabbit, dilution 1:1000 in 5% milk/TBS/0.1% Tween-20;
- PKA alpha Antibody (#PA5-17626; Invitrogen, Waltham, MA, USA), source: rabbit, dilution 1:1000 in 5% milk/TBS/0.1% Tween-20;
- Anti-Na^+^/HCO_3_^−^ Contransporter Polyclonal Antibody (#AB3212-I; EMD Milipore Corporation; Temecula, CA, USA), source: rabbit, dilution 1:500 in 5% milk/TBS/0.1% Tween-20.

The next day, the membranes were washed for 5 × 10 min in wash buffer composed of 1% milk in TBS/0.2% Tween-20, and subsequently incubated with a secondary anti-rabbit antibody (GE Healthcare, Chicago, IL, USA) diluted 1:15,000 in 1% milk in TBS/0.2% Tween-20 for 1 h. Following incubation, the membranes were washed for 3 × 10 min in TBS/0.2% Tween-20 at room temperature and using a stirrer. To visualize the target protein, membranes were incubated with the ECL substrate (GE Healthcare, Chicago, IL, USA) in the dark for 5 min. After incubation, the membranes were placed into the ChemiDoc Imaging System, which automatically calculated the protein visualization time based on the light signal emitted by the membranes [87].

### 4.9. Data Analysis 

For the calculation of relative expression values of the gene expression, we used the delta delta Ct method. The expression of selected genes was determined as the number of amplification cycles obtained from the threshold of the PCR exponential phase. Ct values of biological triplicates were processed in Microsoft Excel for Windows. 

Protein expression was evaluated using BioRad Image Software 6.1 (BioRad, Hercules, CA, USA). 

The results were statistically processed by using the GraphPad Prism program (version 9.4.1 for Mac, GraphPad Software incorporated, San Diego, CA, USA), http://www.graphpad.com/ (accessed on 15 September 2022). For the determination of any significant differences between the control and experimental groups, one-way ANOVA and Tukey’s range test were used. The significance levels were set at *p* < 0.05 (*); *p* < 0.01 (**); *p* < 0.001 (***) and *p* < 0.0001 (****). Data in the graphs are represented as the average values for the individual groups ± SD (standard deviation).

## Figures and Tables

**Table 1:** The sequences of forward-F and reverse-R primers, their accession numbers and their amplicon size in bp (base pairs).

Primer Name	Primer Sequences	Accession Number	Amplicon Size
Bov CATSPER1-F	TACTCTGACCCCAAACGCTT	XM_024987569.1	100 bp
Bov CATSPER1-R	GGCTGTCCAGGTAGATGAGG		
Bov CATSPER2-F	CCTCAAGAGCATGACCTTCC	NM_001192477.1	103 bp
Bov CATSPER2-R	GCGAGTTGAACGGGTGTAAT		
Bov GAPDH-F	GATGGTGAAGGTCGGAGTGAAC	NM_001034034.2	100 bp
Bov GAPDH-R	GTCATTGATGGCGACGATGT		
Bov NBC-F	CAGCCATGACCCACAGGAAT	AF308160.1	146 bp
Bov NBC-R	AGTCTACCTCGCCAACAAGC		
Bov PKA-F	GAAGCCCAAAGCCAGCTCTA	NM_174236.1	109 bp
Bov PKA-R	TTTGATTGAGTCCCAGGCCC		

## Data Availability

The data presented in this study are available upon reasonable request from the corresponding author.

## Supplementary Materials

The following supporting information can be downloaded at: https://www.mdpi.com/article/10.3390/ijms232314646/s1.
