# Supplementary material for: In Vitro versus Cryo-Induced Capacitation of Bovine Spermatozoa, Part 2: Changes in the Expression Patterns of Selected Transmembrane Channels and Protein Kinase A
Source: Int J Mol Sci. 2022 Nov 24;23(23):14646. doi: 10.3390/ijms232314646 (PMC9739406; doi:10.3390/ijms232314646)
Supplement: Supplementary file 1 [file ijms-23-14646-s001.zip › Supplementary Material.pdf]

Supplementary Material

Supplementary Figure S1. Representative photograph of polyacrylamide gels used for the protein separation. 4–15% Mini-PROTEAN TGX Stain-Free Protein Gels (BioRad, Hercules, CA, USA). Picture taken by the ChemiDoc Imaging System (BioRad, BioRad, Hercules, CA, USA).

Supplementary Figure S2. Representative photograph of polyacrylamide gels used for the protein separation. 4–15% Mini-PROTEAN TGX Stain-Free Protein Gels (BioRad, Hercules, CA, USA). Picture taken by the ChemiDoc Imaging System (BioRad, BioRad, Hercules, CA, USA).

Supplementary Figure S3. Original photograph of the CatSper1 blot. PVDF membrane (Trans-Blot Turbo Pack; BioRad, Hercules, CA, USA). Chemiluminescent exposure using the ECL substrate (GE Healthcare, Chicago, IL, USA).

Supplementary Figure S4. Inverted photograph of the CatSper1 blot. PVDF membrane (Trans-Blot Turbo Pack; BioRad, Hercules, CA, USA). Chemiluminescent exposure using the ECL substrate (GE Healthcare, Chicago, IL, USA).

Supplementary Figure S5. Original photograph of the CatSper2 blot. PVDF membrane (Trans-Blot Turbo Pack; BioRad, Hercules, CA, USA). Chemiluminescent exposure using the ECL substrate (GE Healthcare, Chicago, IL, USA).

Supplementary Figure S6. Inverted photograph of the CatSper2 blot. PVDF membrane (Trans-Blot Turbo Pack; BioRad, Hercules, CA, USA). Chemiluminescent exposure using the ECL substrate (GE Healthcare, Chicago, IL, USA).

Supplementary Figure S7. Original photograph of the NBC blot. PVDF membrane (Trans-Blot Turbo Pack; BioRad, Hercules, CA, USA). Chemiluminescent exposure using the ECL substrate (GE Healthcare, Chicago, IL, USA).

Supplementary Figure S8. Inverted photograph of the NBC blot. PVDF membrane (Trans-Blot Turbo Pack; BioRad, Hercules, CA, USA). Chemiluminescent exposure using the ECL substrate (GE Healthcare, Chicago, IL, USA).

Supplementary Figure S9. Original photograph of the PKA blot. PVDF membrane (Trans-Blot Turbo Pack; BioRad, Hercules, CA, USA). Chemiluminescent exposure using the ECL substrate (GE Healthcare, Chicago, IL, USA).

Supplementary Figure S10. Inverted photograph of the PKA blot. PVDF membrane (Trans-Blot Turbo Pack; BioRad, Hercules, CA, USA). Chemiluminescent exposure using the ECL substrate (GE Healthcare, Chicago, IL, USA).

Supplementary Figure S11. Original photograph of the chlortetracycline fluorescence patterns in different stages of the capacitation events. Taken by the Leica DMI6000 B epifluorescent microscope, magnification x 40 (Wetzlar, Germany).
