# Supplementary figures and images for: In Vitro versus Cryo-Induced Capacitation of Bovine Spermatozoa, Part 2: Changes in the Expression Patterns of Selected Transmembrane Channels and Protein Kinase A
Source: Int J Mol Sci. 2022 Nov 24;23(23):14646. doi: 10.3390/ijms232314646 (PMC9739406; doi:10.3390/ijms232314646)

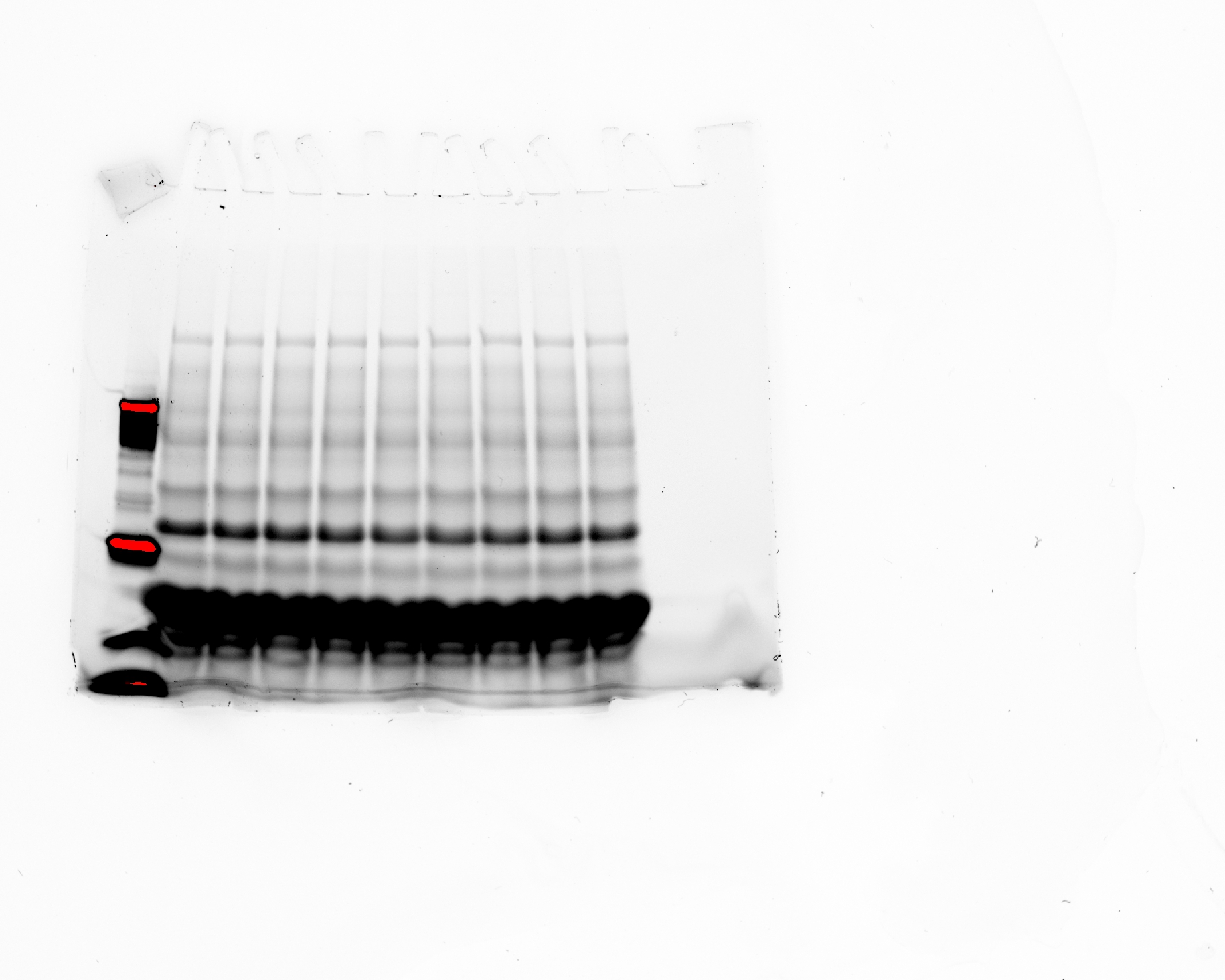

Supplement: Supplementary file 1 [file ijms-23-14646-s001.zip › Supplementary Figure S1.jpg]

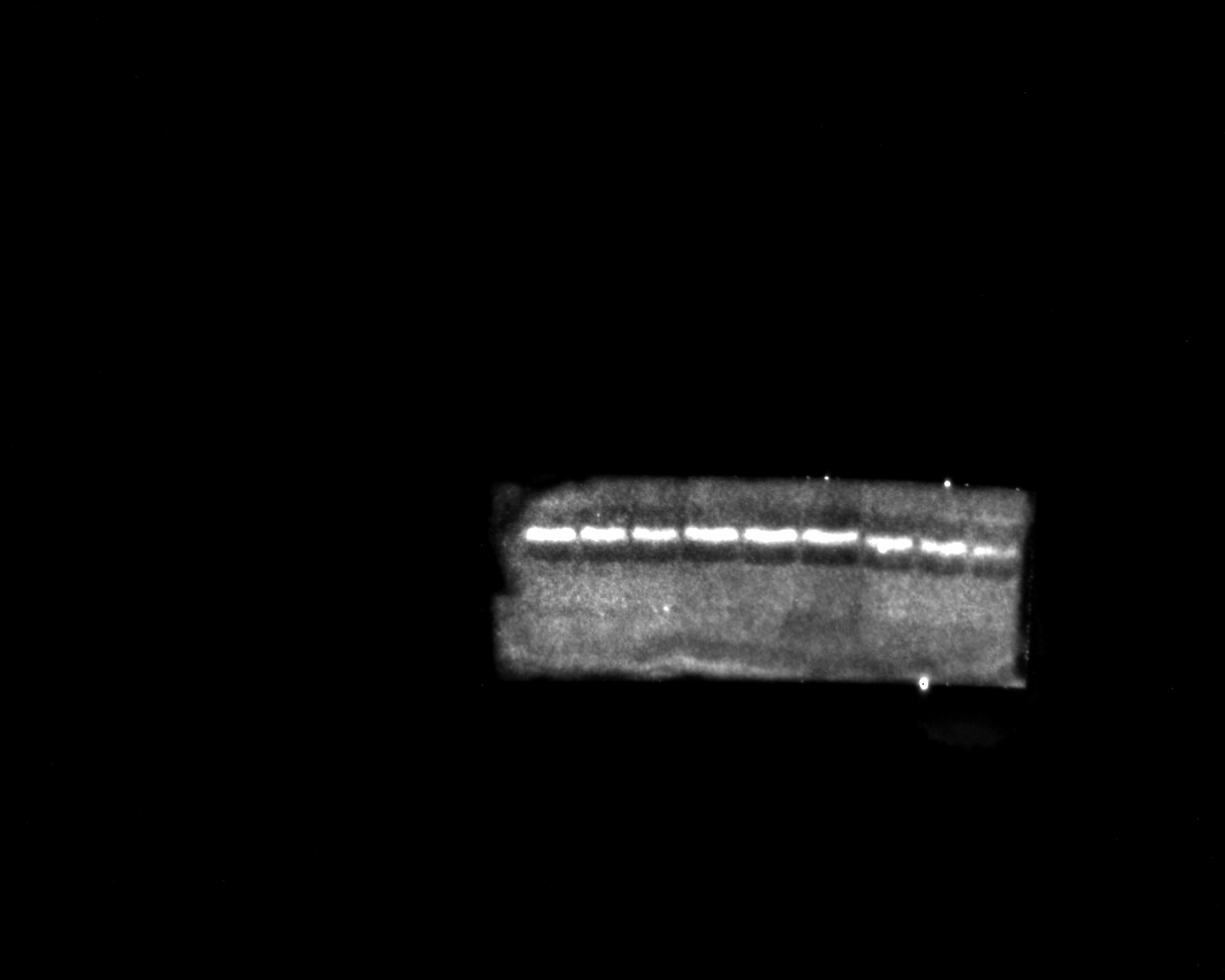

Supplement: Supplementary file 1 [file ijms-23-14646-s001.zip › Supplementary Figure S10.jpg]

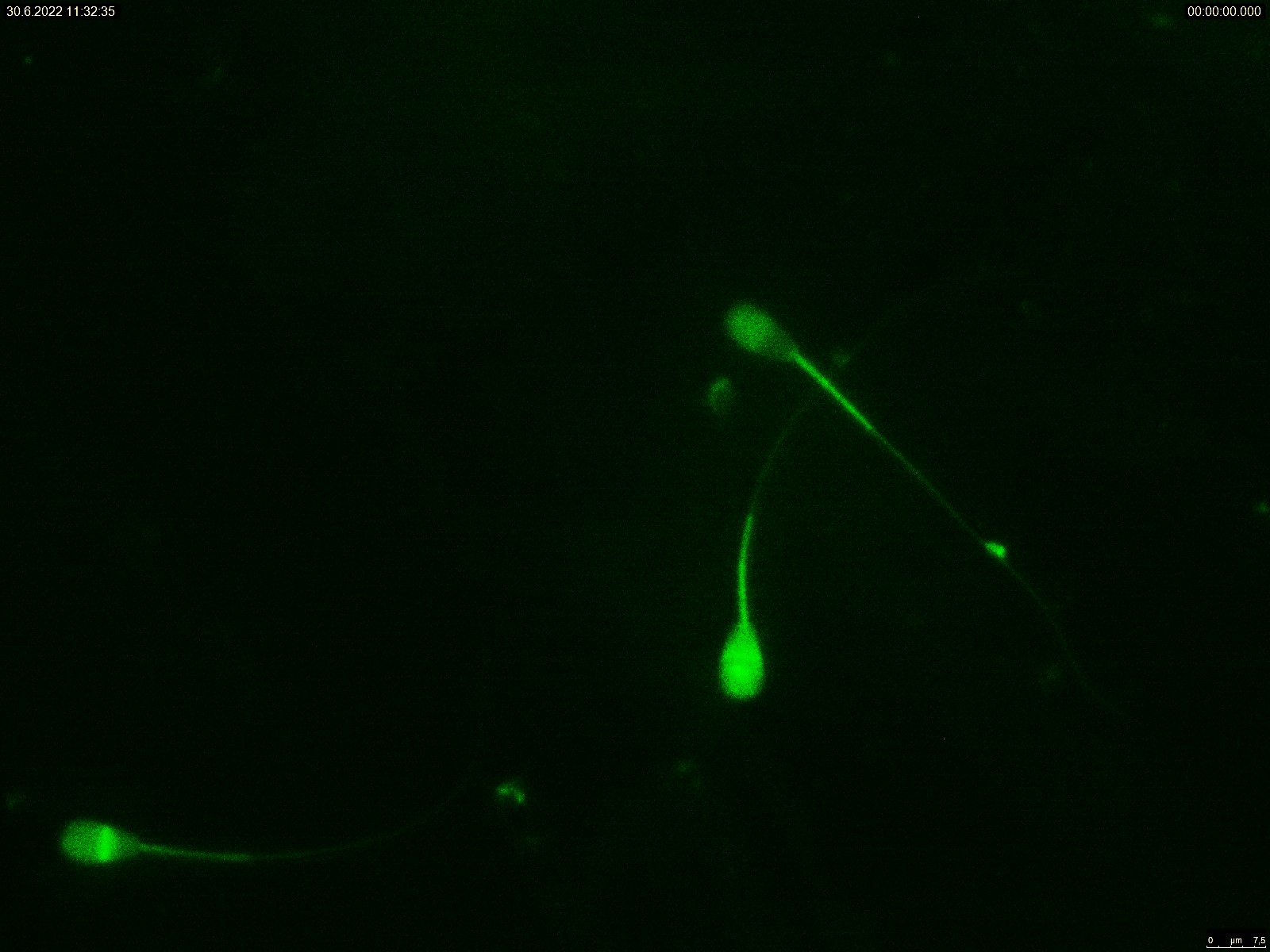

Supplement: Supplementary file 1 [file ijms-23-14646-s001.zip › Supplementary Figure S11.jpg]

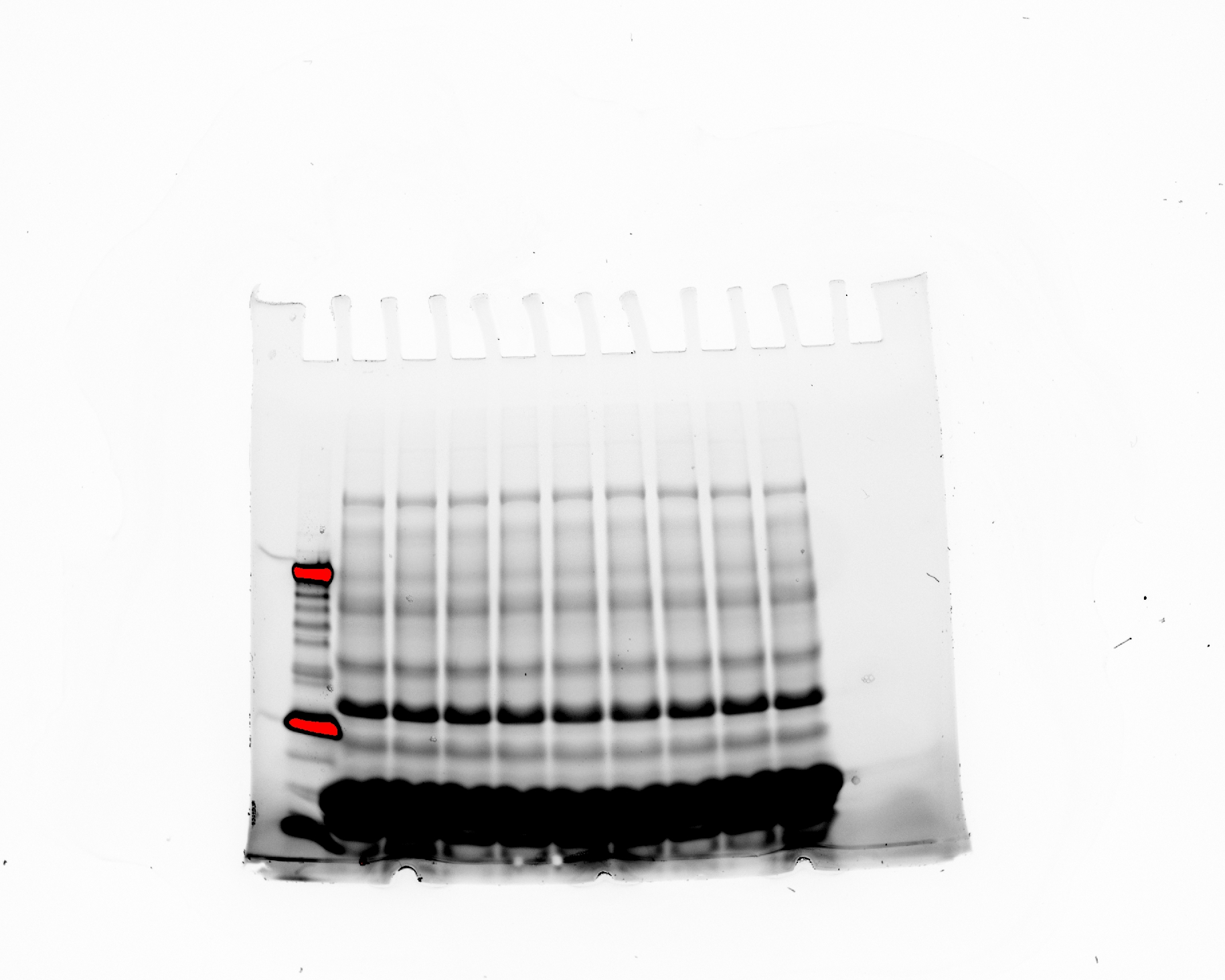

Supplement: Supplementary file 1 [file ijms-23-14646-s001.zip › Supplementary Figure S2.jpg]

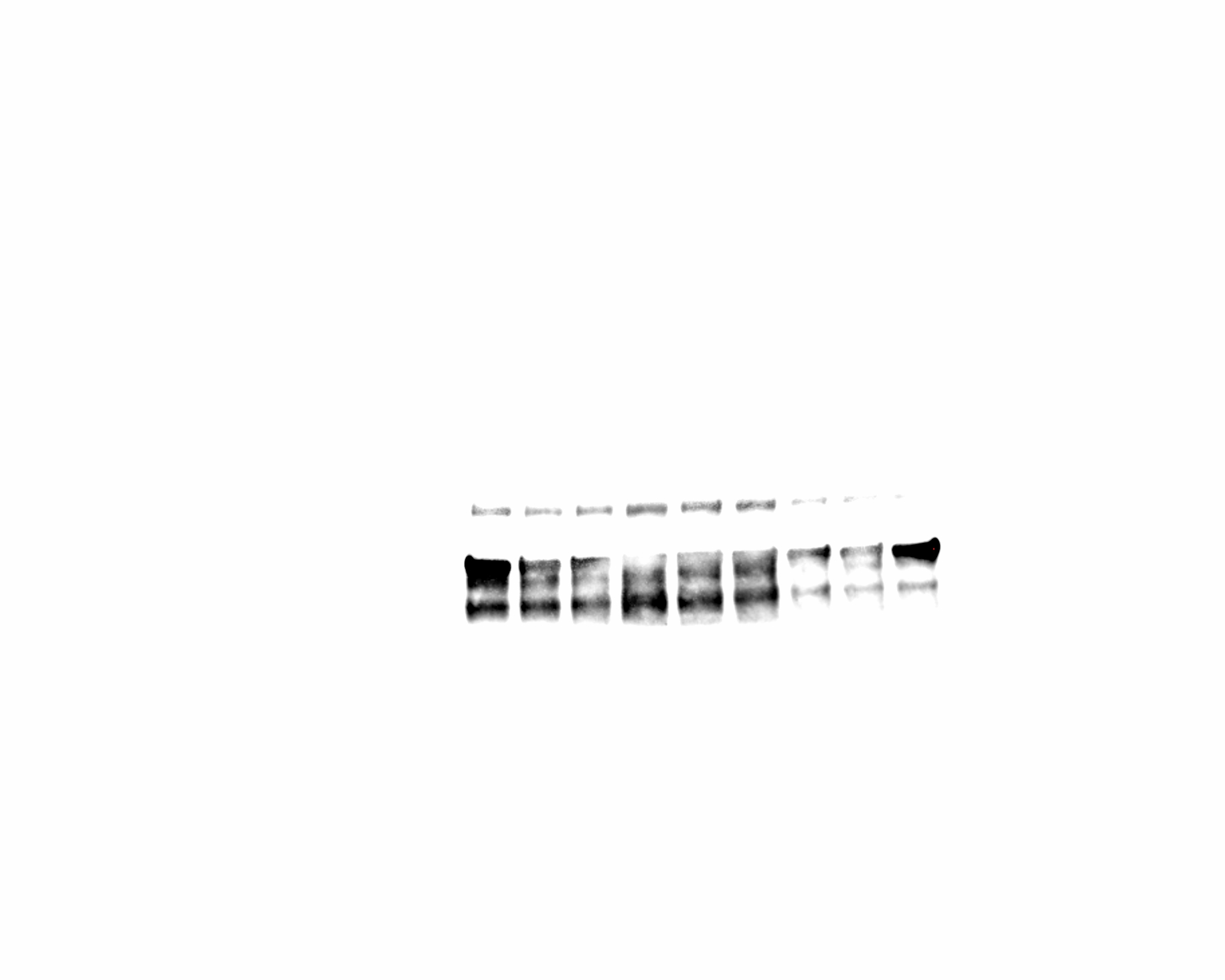

Supplement: Supplementary file 1 [file ijms-23-14646-s001.zip › Supplementary Figure S3.jpg]

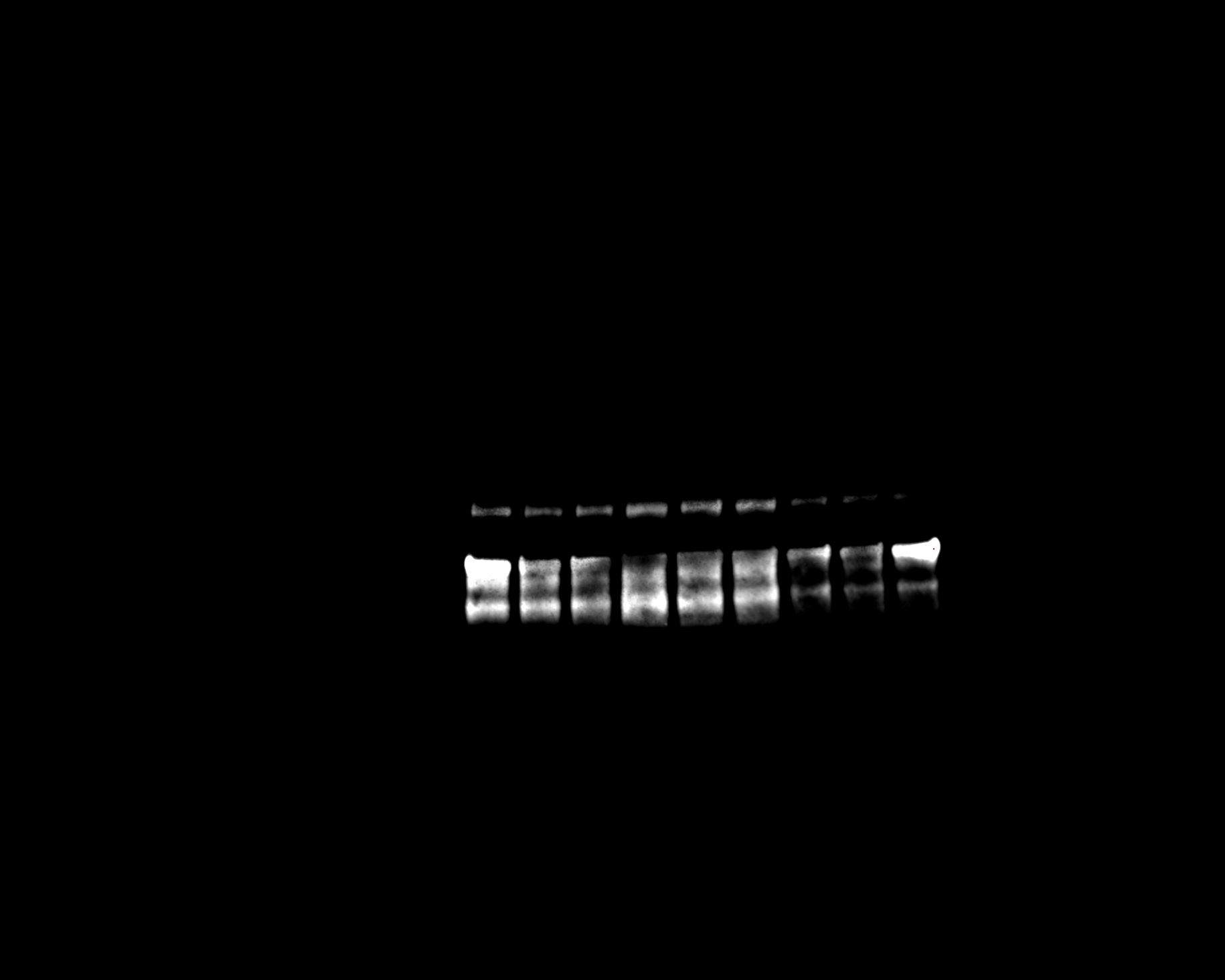

Supplement: Supplementary file 1 [file ijms-23-14646-s001.zip › Supplementary Figure S4.jpg]

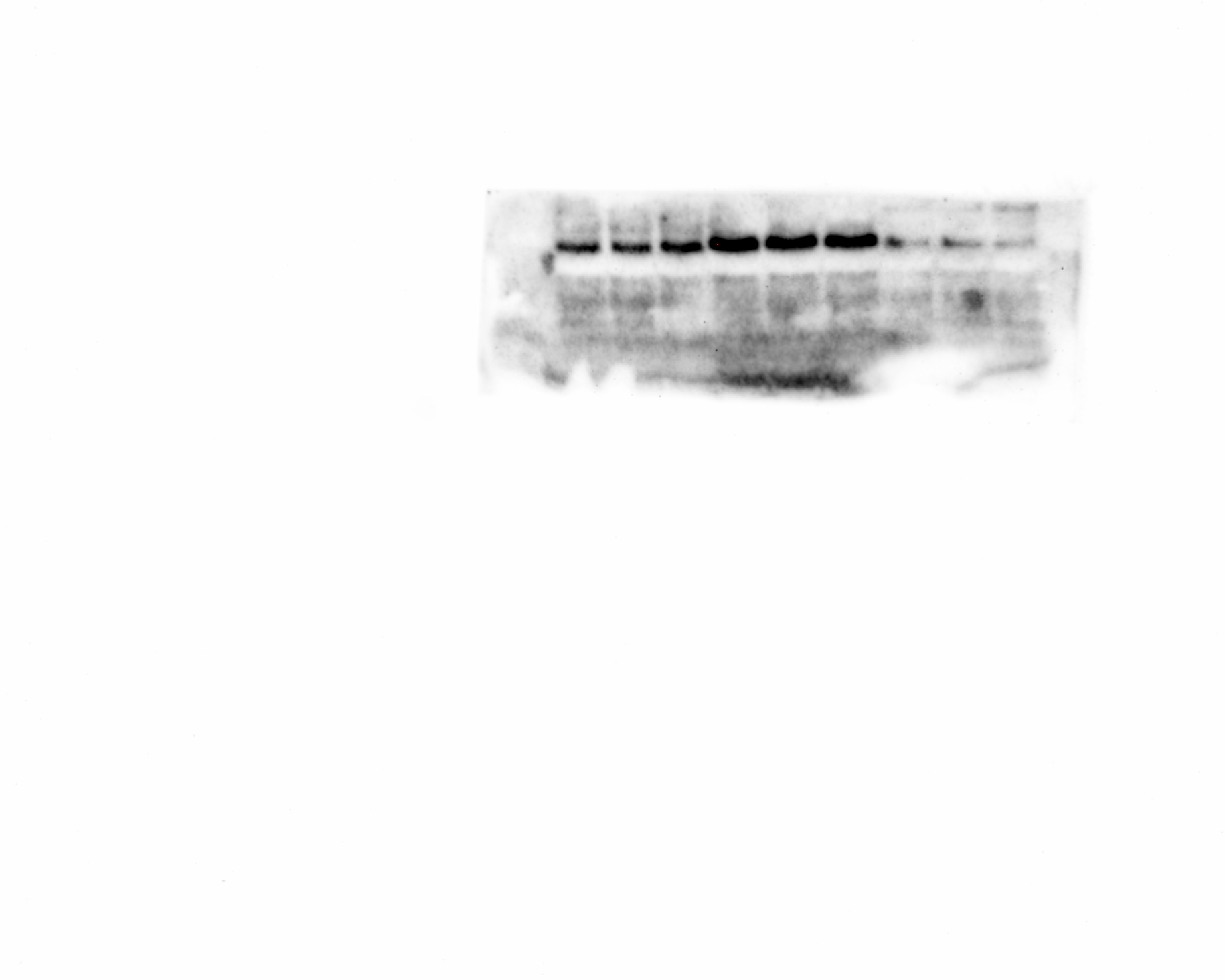

Supplement: Supplementary file 1 [file ijms-23-14646-s001.zip › Supplementary Figure S5.jpg]

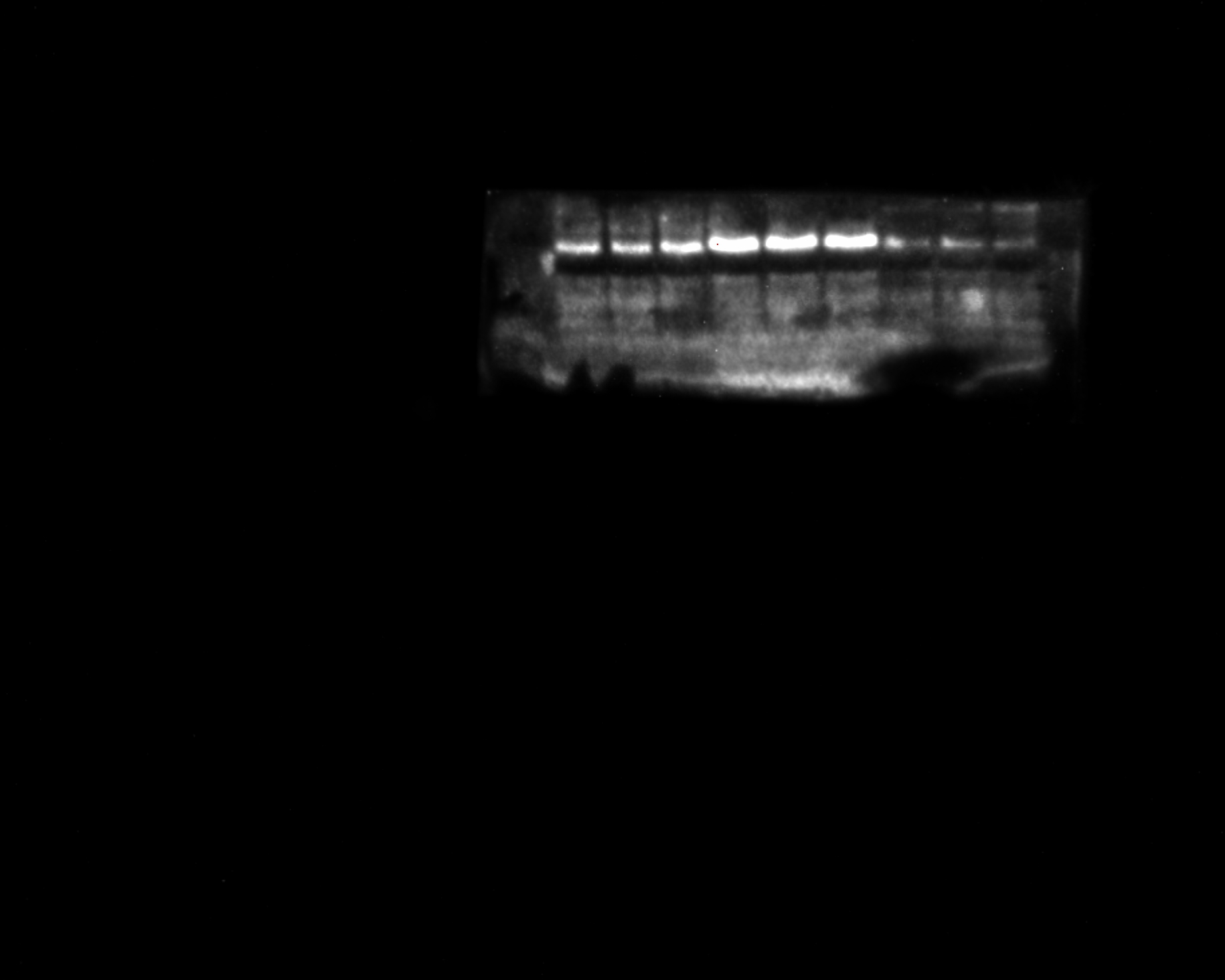

Supplement: Supplementary file 1 [file ijms-23-14646-s001.zip › Supplementary Figure S6.jpg]

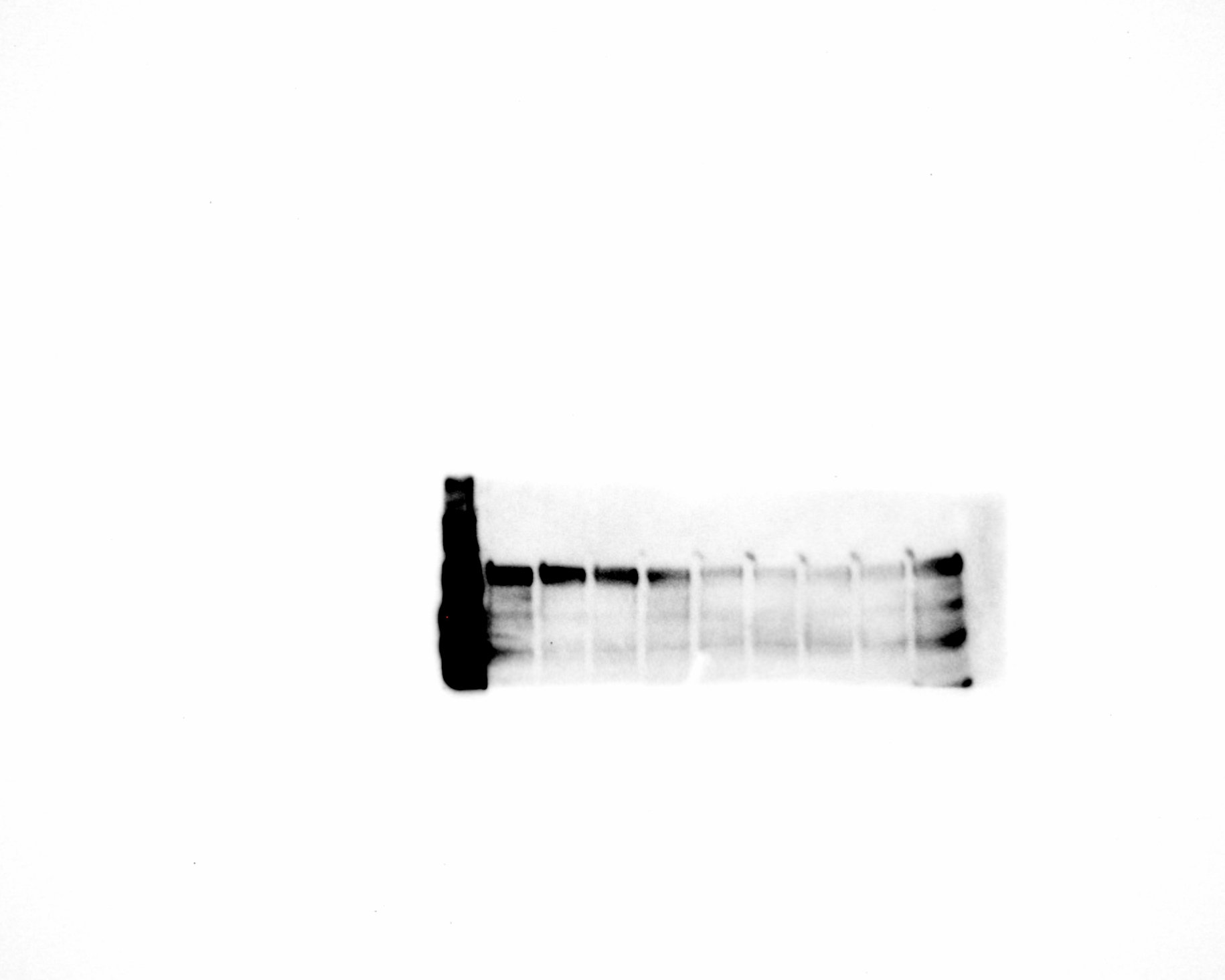

Supplement: Supplementary file 1 [file ijms-23-14646-s001.zip › Supplementary Figure S7.jpg]

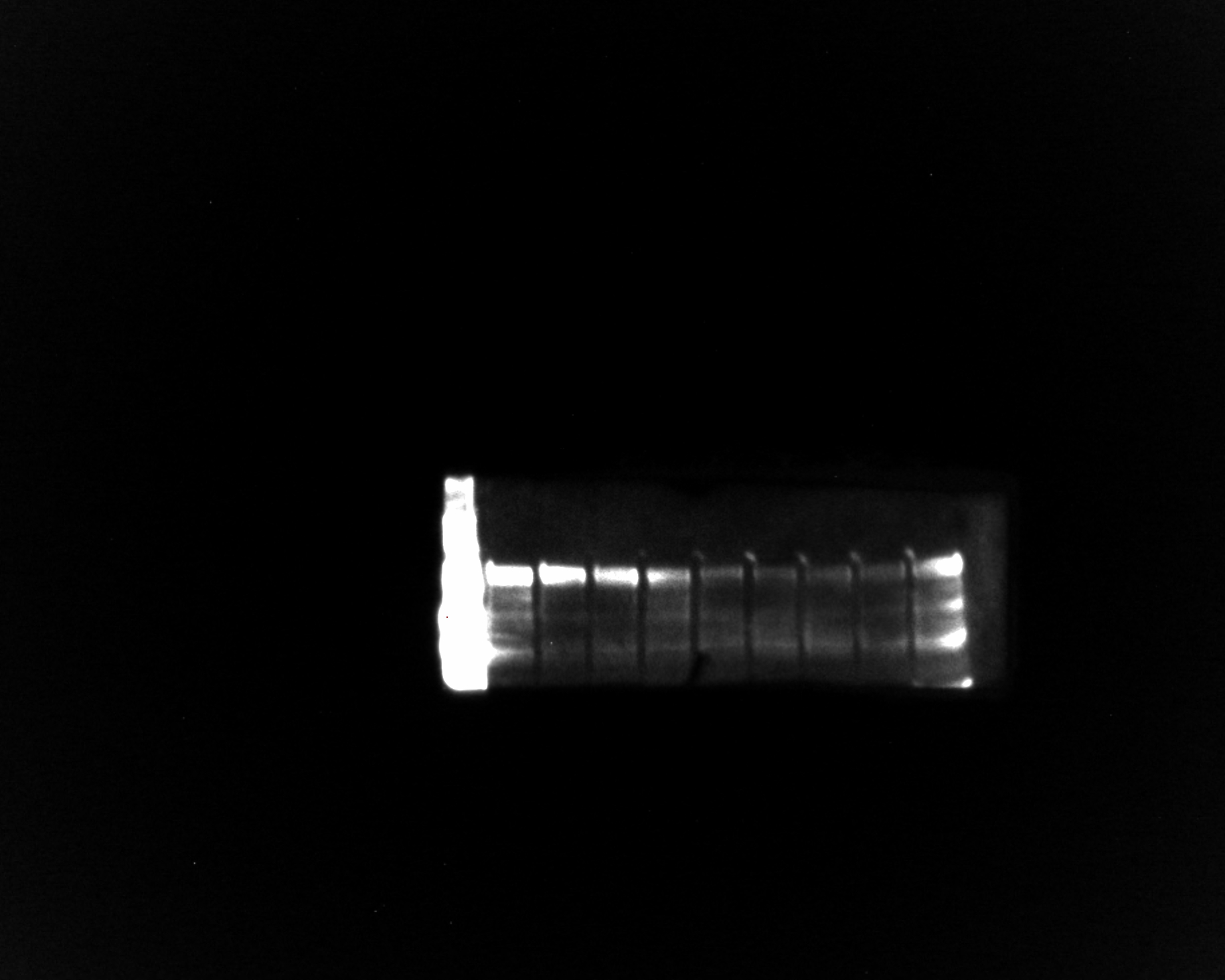

Supplement: Supplementary file 1 [file ijms-23-14646-s001.zip › Supplementary Figure S8.jpg]

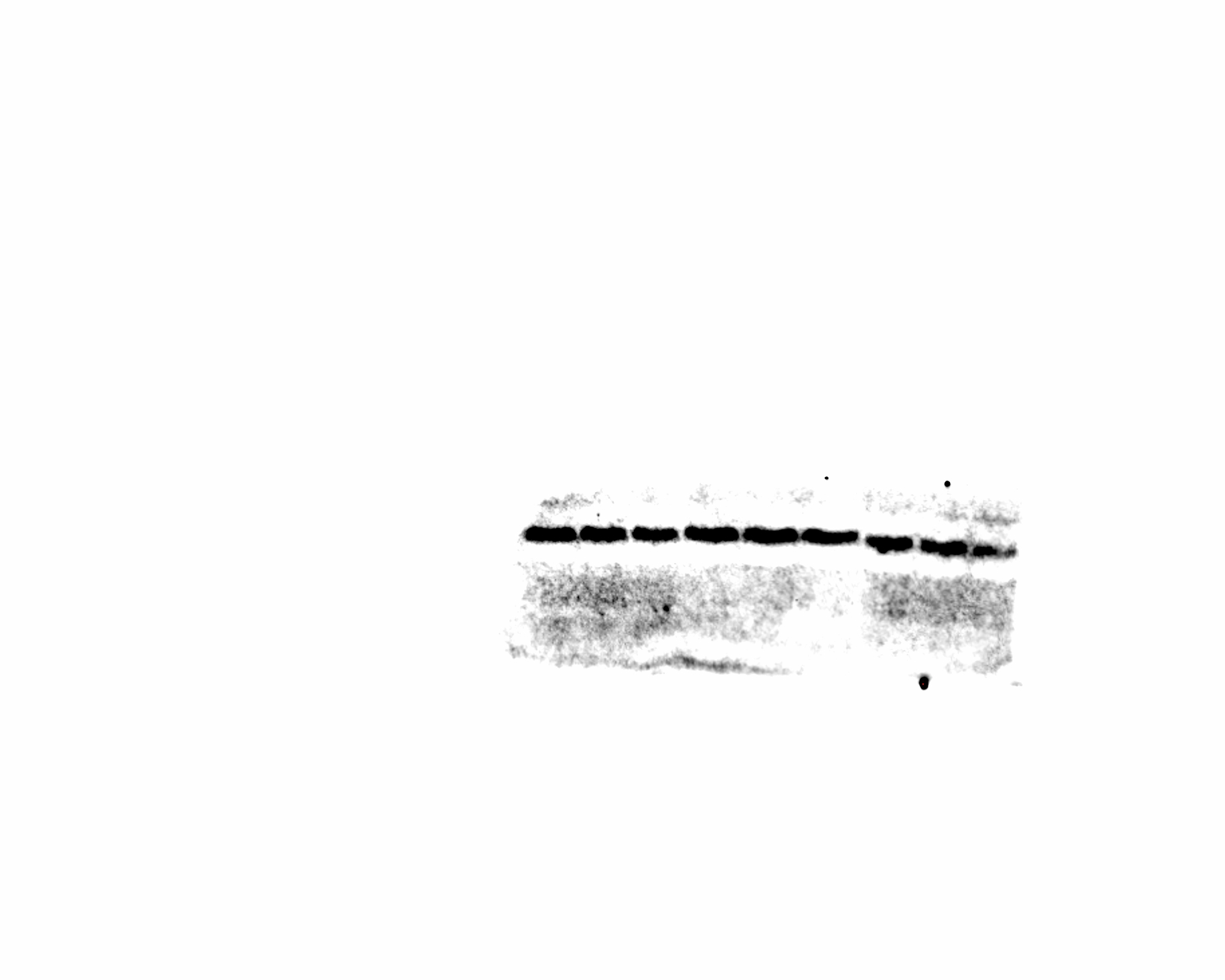

Supplement: Supplementary file 1 [file ijms-23-14646-s001.zip › Supplementary Figure S9.jpg]
